# The emergence and diffusion of DNA microarray technology

**DOI:** 10.1186/1747-5333-1-11

**Published:** 2006-08-22

**Authors:** Tim Lenoir, Eric Giannella

**Affiliations:** 1 Jenkins Collaboratory for New Technologies in Society, Duke University, John Hope Franklin Center, 2204 Erwin Road, Durham, North Carolina 27708-0402, USA

## Abstract

The network model of innovation widely adopted among researchers in the economics of science and technology posits relatively porous boundaries between firms and academic research programs and a bi-directional flow of inventions, personnel, and tacit knowledge between sites of university and industry innovation. Moreover, the model suggests that these bi-directional flows should be considered as mutual stimulation of research and invention in both industry and academe, operating as a positive feedback loop. One side of this bi-directional flow – namely; the flow of inventions into industry through the licensing of university-based technologies – has been well studied; but the reverse phenomenon of the stimulation of university research through the absorption of new directions emanating from industry has yet to be investigated in much detail. We discuss the role of federal funding of academic research in the microarray field, and the multiple pathways through which federally supported development of commercial microarray technologies have transformed core academic research fields.

Our study confirms the picture put forward by several scholars that the open character of networked economies is what makes them truly innovative. In an open system innovations emerge from the network. The emergence and diffusion of microarray technologies we have traced here provides an excellent example of an open system of innovation in action. Whether they originated in a startup company environment that operated like a think-tank, such as Affymax, the research labs of a large firm, such as Agilent, or within a research university, the inventors we have followed drew heavily on knowledge resources from all parts of the network in bringing microarray platforms to light.

Federal funding for high-tech startups and new industrial development was important at several phases in the early history of microarrays, and federal funding of academic researchers using microarrays was fundamental to transforming the research agendas of several fields within academe. The typical story told about the role of federal funding emphasizes the spillovers *from *federally funded academic research *to *industry. Our study shows that the knowledge spillovers worked both ways, with federal funding of non-university research providing the impetus for reshaping the research agendas of several academic fields.

## Background

Since the work of Rosenberg and Kline[1], von Hippel[2], Jaffe[3,4], Trajtenberg[5], and others, economists have abandoned the linear model of innovation which pictured a direct flow of innovation leading from scientific discovery to product development, ending with market introduction of new products. The linear model has been replaced with a model that stresses the role of linkage, feedback, and co-evolution among the various stages of the innovation process from discovery through development to commercialization, and features interdependencies and learning across the various stages of the innovation process. According to this picture, innovation is a dynamic process drawing upon scientific and technical knowledge as well as from manufacturing experience, and insights from business services that provide financing, marketing, regulatory, and commercial knowledge.

Despite the support for the network model of innovation, there have been few (if any) examinations of the impact of industry-based R&D or of the broader technological infrastructure of a region on the research environment of universities. Most examinations of the role of external effects on the university research environment have focused on the impact of defense department funding on science and engineering research during the Cold War era, or on the potential (almost entirely negative) effects of corporate sponsorship of academic research programs in biomedicine. The networked model of innovation described above, however, posits relatively porous boundaries between firms and academic research programs as one key element of an innovative region. The model suggests a bi-directional flow of input between university and industry innovation, in the form of licenses on inventions, personnel, and tacit knowledge flowing from (mostly federally funded) academic research programs, as well as a flow from industry to the universities of new technologies and research directions. Moreover, the model suggests that these bi-directional flows should not be considered as sequential; that is, originating in the university environment and diffusing outward to stimulate commercial innovations that subsequently reshape the academic research environment. Rather, the model suggests the possibility of mutual stimulation of research and invention in both industry and academe, operating as a positive feedback loop.

The flow of inventions into industry through the licensing of university-based technologies has been well studied, and our paper will contribute to that work; but the reverse phenomenon of the stimulation of university research through the absorption of new directions emanating from industry has yet to be investigated in much detail. Our study addresses this issue through the examination of the sources of support, particularly federal support, and the multiple pathways through which commercial microarray technologies have transformed core academic research fields. The first microarray system, the Affymetrix GeneChip^® ^originated beyond the walls of the academy, but within a decade it made significant inroads into reshaping the research environments of university programs as well as launching a spectrum of competitive firms in several industrial sectors within the Silicon Valley and other high technology regions. Academic researchers collaborating with Affymetrix scientists were quick to explore the power of gene chips. They sought to improve upon and adapt gene chips being supplied by firms such as Affymetrix to their research questions. In addition, several academic researchers connected with the Human Genome Initiative actively pursued development of alternative types of DNA microarrays, particularly spotted and ink-jet microarrays, as competitor systems to the GeneChip^®^. While many of the university-based microarray systems were assembled in-house as home brew systems, several found their way into industrial development. Since the mid-1990s the lively – sometimes legally disputed – competition between these platforms deemed essential for developing a more systemic understanding of genetics has been responsible for attracting hundreds of millions of dollars into biotechnology and pharmaceutical companies. Following initial application in combinatorial synthesis of organic materials, most spectacularly implemented as the original Affymetrix GeneChip^® ^in 1994, microarrays drawing upon concepts of the original biochips were developed for combinatorial materials synthesis of inorganic materials as well. By early 2000 the sky seemed to be the limit for all branches of microarray technology.

The broad, significant impact and continuing rapid enhancement of microarrays make the technology a suitable "probe" for tracking the various functions of different types of institutions in the diffusion of an important technology. These institutions include the federal government, universities and non-profit research institutions, startups, established companies, and business services such as legal firms and venture capital firms. We want to understand the nature of institutional interactions in the case of DNA chips and which relationships were particularly crucial to advancing the technology as a major platform in biomedical discovery. We will focus on the story of microarrays from a variety of angles: we examine the impetus for organizations or groups of researchers to become involved with microarrays, the contextual factors that enabled their participation, and how they applied their existing expertise and collaborated with others to use microarrays or build related systems. And finally, we trace how these innovators' work contributed to changing the overall landscape of research.

## Results and discussion

### 1. The invention of the GeneChip^®^

The microarray and gene chip grew out of efforts by a team of scientists concerned with optimizing methods of drug discovery. This group was assembled by Alex Zaffaroni, the legendary CEO of Syntex and later founder of several biotech firms, including Alza and DNAX. In 1988 Zaffaroni approached Lubert Stryer, professor of biochemistry at Stanford and inventor of numerous fluorescence-tagging methods for enabling the Fluorescence Activated Cell Sorter (FACS) as one of the primary tools of cell biological research, to become the chief scientific officer of the new company Zaffaroni, J. Leighton Read, and Peter Schultz [Note A] were founding called Affymax. The goal of Affymax was to develop novel chemical approaches to automated drug discovery. The traditional approach in drug discovery had been to synthesize or discover new candidate drugs and then test their activities one at a time. This is a tedious, cumbersome, and increasingly expensive approach, so speeding up or automating this process was of substantial interest to pharmaceutical companies.

In building the company, Zaffaroni, Schultz, and Read did not have a specific technology they intended to pursue. However, feeling that recent developments in biotechnology were about to render the problem of drug discovery tractable, they assembled a star-studded scientific advisory board from Stanford and several other universities [Note B]. From the beginning the approach advocated by Avram Goldstein of the Stanford Pharmacology Department seemed most appealing. Goldstein urged the pursuit of peptide synthesis as a means of generating chemical diversity for identifying promising leads for drug molecules. Goldstein argued that since receptors for any ligand can be formed from short peptide sequences in the combining sites of antibodies, it must be conversely true that from short peptides, one could make a ligand for any receptor [6]. Affymax could pursue the generation of large libraries of small peptides with novel sequences against various protein targets, analogous to the way in which the immune system operates by mass screening its antibody repertoire, identifying the ones that work best and making more of those.

Several methods for generating large peptide libraries through what was being called "combinatorial chemistry" were coming on the scene in the mid-1980s. The field actually got its start in 1963, when R. Bruce Merrifield (Nobel Prize in chemistry, 1984) introduced the concept of solid phase peptide synthesis (SPPS) whereby polypeptide chains as short as two amino acids (dipeptides) as well as longer (protein) chains could be made in assembly-line fashion using automated peptide synthesizers. In the 1980s, Australian researcher Mario Geysen of the University of Melbourne (later at Glaxo Wellcome) showed that SPPS could be the basis of multiple peptide synthesis. Geysen's "peptide on a pin" method generated a variety of short protein fragments by combining multiple amino acids (the building blocks of peptides and proteins) in different permutations [7,8]. Each peptide was made on the end of a pin-shaped polyethylene support, dipped into a dish with a new amino acid for each step in the reaction. By lining the pins in an array with the (originally 96) wells of a microtitre plate dozens (even hundreds) of reactions could be performed at the same time. Geysen's method was the first example of a library of synthesized compounds where the molecular identity could be known based on the physical position of the compound in the library [Note C].

Other candidate techniques for generating combinatorial libraries of peptides were coming on the scene at about the same time the Affymax board was developing its approach [Note D]. But rather than pursuing any of these options in developing combinatorial syntheses, Read and Pirrung came up with a brilliant new approach of their own which they called VLSIPS, for Very Large Scale Immobilized Polymer Synthesis. In one of the meetings of the Affymax scientific board, Leighton Read tossed out the idea of just mimicking the makers of semiconductor chips, who use beams of light to manipulate molecules on solid surfaces in order to create random chemical diversity. Though he had spent his career working with light activation and fluorescent labeling, Stryer had not thought of this possibility. Pirrung and Read got to work on the idea and wrote up an invention record on VLSIPS, modeling the name on the VLSI (very large scale integration) technology that was driving the semiconductor industry at the time [Note E]. Read and Pirrung defined the concepts and major parameters of light-directed synthesis over the next few days, which they detailed in a patent application filed on June 7, 1989.

The next step for the group was to begin work on implementing the idea of generating chemical diversity on an array designed by a photolithographic process. Pirrung was about to head off to Duke University to take up a new professorship in biochemistry, so Stryer began inquiring among local colleagues for the name of a young biochemist who might be appropriate to head up the project of producing a prototype and reducing the invention to practice. Stryer's long-time Berkeley collaborator Alexander Glazer suggested Stephen Fodor, a young Princeton Ph.D. with a NIH postdoctoral fellowship working on time-resolved spectroscopy of bacterial and plant pigments in his lab. Glazer recommended Fodor as a biochemist of exceptional ability; indeed, he already had the reputation of a visionary. Although taking a position in industry was not of interest to Fodor, the opportunity to brainstorm with Zaffaroni, Stryer, Berg, Schultz, Lederberg and Davis was an opportunity he did not want to miss. The academic appointment could follow. In July of 1989 Fodor joined the group at the Affymax offices on Porter Drive in the Stanford Industrial Park.

**Figure 1:**
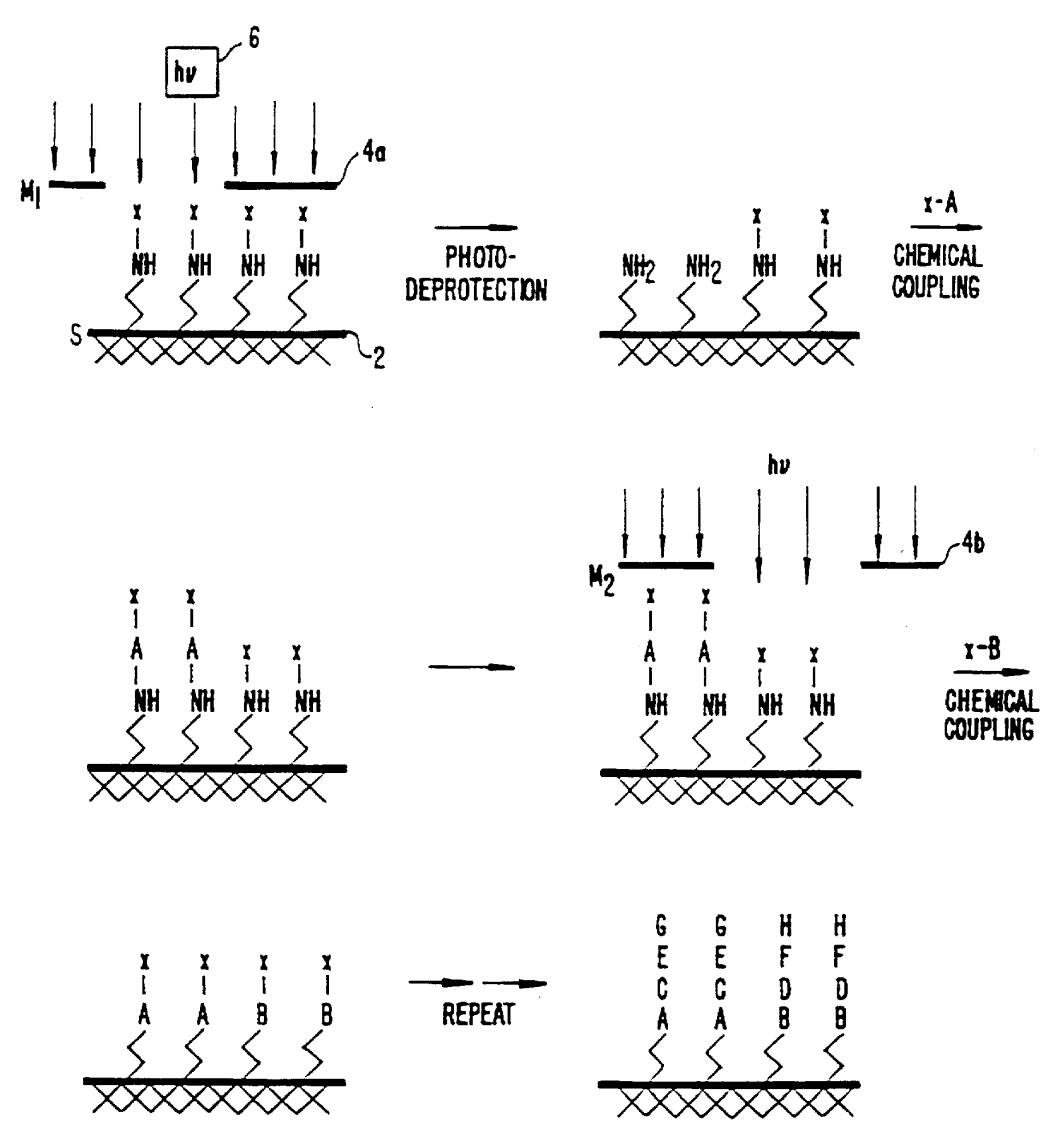
**Concept of Light-Directed Spatially Addressable Parallel Chemical Synthesis**. **Source**: Fodor SPA, Stryer L, Read JL, Pirrung MC: **USPTO 5,744,305**. Arrays of Materials Attached to a Substrate, April 28, 1998, Sheet 1.

Over the next 18 months Fodor worked intensely with Stryer in what both describe as the most stimulating and productive period of scientific invention imaginable. The invention they, together with their scientific colleagues at Affymax, ultimately produced – light-directed spatially addressable chemical synthesis – was quite literally the marriage of biochemistry and the photolithography techniques used in chip design in the local semiconductor industry. What they demonstrated by 1991 in a now classic article published in *Science *was a process for depositing onto a glass substrate – literally a microscope slide cover in the first version of the invention – amino acid groups – NH – that were blocked by a photolabile protecting chemical group – X (Figure 1) [9]. Illumination with a laser through a mask led to photodeprotection, allowing, in the next step through chemical coupling, the addition of a first chemical building block A containing a photolabile protecting group X. In the next step, a different mask is used to photoactivate a different region of the substrate. A second labeled group B is then attached to the amino groups exposed by the illumination through the mask. This process is repeated as many times as desired to obtain the desired set of products. By washing a bath of peptide chains with a fluorescent marker attached to the end, it was possible to determine the composition of amino acids forming the chain with the aid of a photomultiplier/scanner that operated similarly to the FACS (Fluorescence Activated Cell Sorter). The initial microarray consisted of 1024 peptides in a 1.6 cm^2 ^area generated in a ten-step process. This was the first microarray designed specifically for peptide synthesis, and at the same time Fodor developed a scanner for reading the output.

Part of the beauty of combining photolithography with combinatorial chemistry is the resultant high density of the compounds on the substrate. Theoretically, the only physical limitation on the density is the degree to which the compounds can be activated – in other words, the diffraction of light. This provides for an incredibly high degree of miniaturization, and in 1991, at the time of the publication of the paper, Fodor and his colleagues at Affymax wrote:

Our present capability for high-contrast photodeprotection is better than 20 μm, which gives >250,000 synthesis sites per square centimeter. There is no physical reason why higher densities of synthesis sites cannot be achieved [10].

Using photolithography essentially brought Moore's Law to Affymax and to the new company Fodor launched around it, Affymetrix. Similarly to the semiconductor industry, Affymetrix has steadily increased the density of synthesis sites, while making the chips more complex and harder to manufacture [11]. Indeed, five years later, Affymetrix had produced a prototype chip with a million probes [12].

While work continued on peptide microarrays, Stryer and the Affymax scientific board recognized a much more immediate opportunity in the development of nucleic acid microarrays. Solid phase synthesis of DNA is considered the most effective and reliable method of chemical synthesis known. Given the strict base-pairing rules (Watson-Crick pairing) obeyed by the four building blocks of DNA (adenine, cytosine, guanine, and thymine, or A, C, G, and T), a section of single-stranded DNA, which might contain numerous genes and thus be used as a probe, will match up only with its complementary strand of DNA [cDNA for "complementary DNA"] to form the double helix. RNA, which is DNA's chemical cousin, also follows a strict base-pairing rule when binding to DNA, so the sequence of any RNA strand that pairs up with DNA on a microarray can be inferred as well.

Combinatorial analysis based on light-directed synthesis of DNA on a chip offered excellent opportunities, and there were a number of reasons why Fodor wanted to pursue DNA chips more vigorously than peptide arrays. Simply in terms of practical considerations of construction, a peptide array of just two amino acid units for each sequence with the 20 amino acids as building blocks produces an array of 400 sequences in 40 steps of the sort described above. By contrast, in the same number of steps (40), a DNA array of 10 unit (ATTGC...) sequences each synthesized from the four nucleic acids as building blocks can be constructed containing an array of one million sequences. Moreover, DNA was ideally suited for light-directed synthesis, and well-established techniques existed for anchoring the DNA to a glass plate. Having demonstrated that light-directed synthesis of peptides using photolithographic masking technology was possible, and knowing that all the pieces for doing a parallel DNA synthesis were within reach, Fodor was eager to shift his attention entirely to developing the gene chip. In a fax of May 15, 1990 to Stryer, Fodor outlined the reasons for his convictions that it was time to devote full concentration on the gene chip. The upshot of this was to spin off the gene chip project as its own company, Affymetrix (for Affinity Matrix).

### 2. Drawing on the Silicon Valley Network

Fodor's prototype of the light-directed parallel peptide synthesis array, the fluorescence scanner and computer system for keeping track of each spot on the chip and quantifying the ratio of tagged DNA matches as color spot ratios in a computer graphic output, the photomasks and appropriate photochemicals for constructing the arrays were all designed in the heady "think tank" environment of Affymax. The discussions in the Affymax scientific board meetings allowed Fodor and his colleagues to draw upon the knowledge and vision of some of the leading academic biochemists and chemists of the day from several universities, including Stanford, Berkeley, Cal Tech, and Lawrence Livermore Labs. This back-and-forth flow of information between academic researchers and the efforts to launch the company had very much the style and spirit of a Silicon Valley startup.

The distributed networked character of innovation in the Silicon Valley is exemplified by Fodor's original prototype system for using photolithography to design a peptide microarray. As a way to construct chemical diversity, Stryer suggested laying down a grid of parallel stripes – each stripe with a different compound – one compound at a time, then repeating the procedure with stripes laid down perpendicularly on the grid, one at a time. In order to see how to work it out using photolithography, Fodor contacted Fabian Pease, professor of electrical engineering at Stanford, specializing in electron beam lithographic mask fabrication. Pease, a Ph.D. from Cambridge University, had been an assistant professor at UC Berkeley briefly before moving, in 1967, to Bell Labs, where he first worked on digital television and later led a group that developed the processes for electron beam lithographic mask manufacture and demonstrated a pioneering LSI circuit built with electron-beam lithography. Pease had been at Stanford since 1978. Fodor and Stryer persuaded Pease to join Affymax as a consultant on their project, and he and Fodor spent a lot of time discussing technical aspects of lithography needed to build the microarray. Pease took Fodor around to various warehouses in Silicon Valley to acquire old lithography instruments needed for building the prototype peptide array. By May, 1990 with periodic input from Pease, Fodor had a working semi-automated lithography instrument that would do binary combinatorial peptide syntheses. Pease maintained his connection to Fodor after the launch of Affymetrix in 1992. In 1993–94, for instance, he took a sabbatical from Stanford to work on the DNA microarray. Pease has been co-inventor along with Fodor and Stryer on several key Affymetrix patents, and he has continued to maintain a consulting relationship with Affymetrix [Note F].

A similar story of Silicon Valley networking led to the design of the first microarray scanner and reader. Through the network of contacts of the Affymax scientific board, Fodor got in touch with Peter Fiekowsky to assist him in the development of a system for detecting and imaging the fluorescently labeled markers of polymer sequences on the peptide array. Fiekowsky had received his BS degree in physics from MIT in 1977. Following graduation, he moved to Silicon Valley to work at NASA and moved to Fairchild's artificial intelligence lab in 1983, where he worked on image analysis in the semiconductor industry. A year later, in 1984, Fiekowsky founded Automated Visual Inspection. The work he and Fodor did on the array project led to two of the 23 patents Fiekowsky holds in image-processing techniques ranging from semiconductor and flat panel inspection to medical x-rays and gene chips [13,14].

James L. Winkler's involvement with Fodor and the core technologies in the launch of Affymetrix provides another typical example of the wide range of talents and the veritable gene pool of innovators who circulate through startups in Silicon Valley. As Fodor recalled in an interview, "Winkler was one of these guys who was just brilliant, did not have any formal education, but could build anything. He could take a blank circuit board and by the end of the day have something he could plug into the back of the computer to run an external piece of equipment." [Interview with Stephen Fodor, August 2004] One of Winkler's first contributions was the design and implementation of the method and devices for flowing reagents through block channels on the glass microarray substrate to form the stripes of different peptides in combination with the light-directed method of coupling and decoupling. After each stripe was laid down, the substrate was shifted by a rotating stage, and the process repeated to form arrays of polymers on the substrate [14]. This was just the first of what would become 31 patents on different aspects of gene chip production and photolithographic mask design, including a set of computer tools for selecting probes and designing the layout of an array of DNA or other polymers and using chip design files to design and/or generate lithographic masks [16].

The guidance that Affymetrix received in its nascent years from consultation arrangements with academics and other local Silicon Valley experts was crucial to the advance of gene chips and related systems. Research in several domains had been going on for years in university and government research projects that provided fertile sources of ideas and techniques for developing the complex technology of the DNA microarray. In fact, university scientists appeared several dozen times on granted Affymetrix patents, although some of these can be accounted for by university faculty who had been hired into the company [Note G]. In Table 1 we present the results of our scan of the patent data for academic collaborations with Affymetrix [Note H].

**Table 1:** Some of the University Faculty Appearing on Affymetrix Patents

**Institution**	**Collaborator**	**Department – General Research Area**
Stanford	Stryer; Lubert	School of Medicine – Biochemistry
	Davis; Ronald W.	School of Medicine – Biochemistry and Genetics
	Pirrung; Michael C.	Department of Chemistry – Organic Chemistry
	Pease; R. Fabian	Department of Electrical Engineering – Semiconductor Manufacturing
	Quate; Calvin F.	Department of Electrical Engineering – Nanomanufacturing
Princeton	Levine; Arnold J.	Department of Biochemistry – Oncology
University of California	Mathales; Richard A.	Department of Chemistry – Biophysical Chemistry
	Schultz; Peter G.	Department of Chemistry – Biochemistry
Argonne National Laboratory	Mirzabekov; Andrei	Biochip Technology Center – Molecular Biophysics
University of Michigan	Collins; Francis S.	Department of Internal Medicine – Human Genetics

We believe that these university collaborators provided enabling expertise to Affymetrix, without these ongoing consultations the development of the microarray would have taken much longer. Federal funding has been particularly important in the development of microarrays. On the one hand, as we have seen, federal funding for extra-university-based industrial research and development provided the capital to launch the cluster of innovative technologies directly connected with the GeneChip^® ^at Affymetrix; and as we shall show in our case studies further on, federal funding was crucial for the take off of some competitor technologies in the microarray field. But the work at Affymetrix and other firms in the microarray field was heavily dependent on knowledge and expertise that had accumulated in several academic disciplines, including biochemistry, genetics, electrical engineering, and computer science as a result of at least two decades of federal funding from the NSF, NIH, DOE, and programs such as the Human Genome Initiative, particularly at Bay Area universities, Stanford, UC Berkeley, and UCSF. In terms of the infrastructure of innovation discussed above in our introduction, "knowledge spillovers" from these federally supported academic research programs provided important resources to the nascent field of microarrays. Federal funding of extra-university research and development by industry provided the stimulus for drawing those resources into an accumulating ensemble of innovations that gave rise to a major new technology and several new lines of research. Government support, particularly to nearby universities, lowered the cost of development through the cultivation of experts who played a pivotal role in the creation of the GeneChip^®^.

### 3. Federal funding of research and development

The development of combinatorial chemistry, microarrays, and the GeneChip^® ^at Affymax and Affymetrix and other Bay Area companies calls attention to an important but often overlooked feature of the development of high technology regions: namely, the role of federal funding for research and development in companies that transforms the academic research environment while launching new industrial sectors. Most discussions of federal funding for research concentrate on the role of federal funding in driving academic research. But as our analysis of the rise of Affymetrix demonstrates, federal funding has also been crucial in stimulating the other side of the equation in the symbiosis of Silicon Valley and research universities such as Stanford: namely, in the formation of the startup companies and collaborations with large established companies in the development of new innovative technology. We frequently point to the massively central role of the federal government in funding academic research, but it is also the case that in Silicon Valley the government has played and continues to play a large and absolutely vital role in funding new industrial development. This point has been made frequently about the role of defense contracting in support of early developments in the electronics and semiconductor industries during the 1950s–70s. But federal funding has also been a major factor in the development of biotech, materials science, and several related industries from the 1990s to the present.

**Figure 2:**
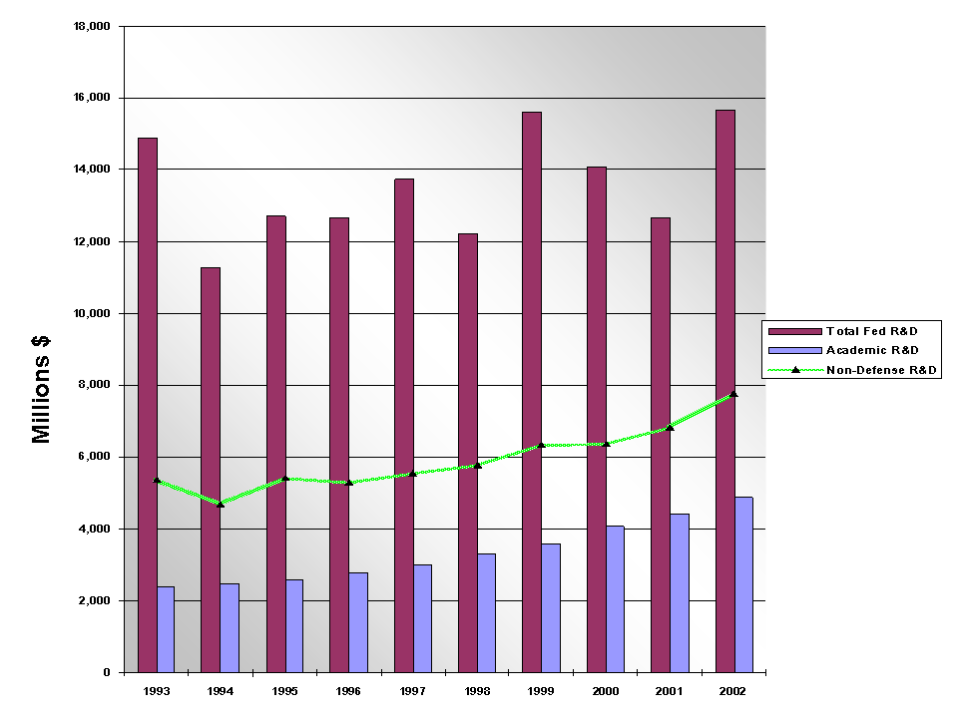
Federal Funding for R&D to California Industrial Firms and to Universities (in millions of dollars).

Table 2 and Figure 2 illustrate the significant contribution of federal funding of both university and industry R&D in California for the period of the 1990s to 2002.

**Table 2:** Federal Funding of R&D in California

**Federal Funding of R&D in California**										
Year	1993	1994	1995	1996	1997	1998	1999	2000	2001	2002
R&D obligations (millions of dollars)	14,884	11,280	12,704	12,658	13,731	12,222	15,600	14,083	12,651	15,686
										
Industry R&D (millions of dollars)	26,541	28,541	28,710	28,710	34,011	35,568	39,047	45,769	41,745	39,664
Academic R&D (millions of dollars)	2,380	2,484	2,594	2,791	2,979	3,302	3,573	4,053	4,422	4,882
life sciences (percent)	58.00%	58.00%	57.00%	56.00%	56.00%	57.00%	56.00%	58.00%	58.00%	58.17%
engineering (percent)	13.00%	13.00%	13.00%	14.00%	15.00%	15.00%	15.00%	15.00%	13.00%	13.08%
physical sciences (percent)	13.00%	12.00%	12.00%	13.00%	13.00%	12.00%	12.00%	12.00%	11.00%	10.63%
Number of SBIR awards*	850	1,012	968	971	1,046	937	992	953	1,036	1,236
Utility patents issued to state residents	8,958	9,263	10,473	11,290	15,793	16,774	17,492	ND	18,598	18,829
Department of Defense (millions of dollars)	9,525	6,598	7,272	7,798	8,171	6,437	9,252	7,717	5,822	7,915

*In addition to SBIR and STTR awards, California firms received 184 ATP awards from 1990–2004

**Figure 3:**
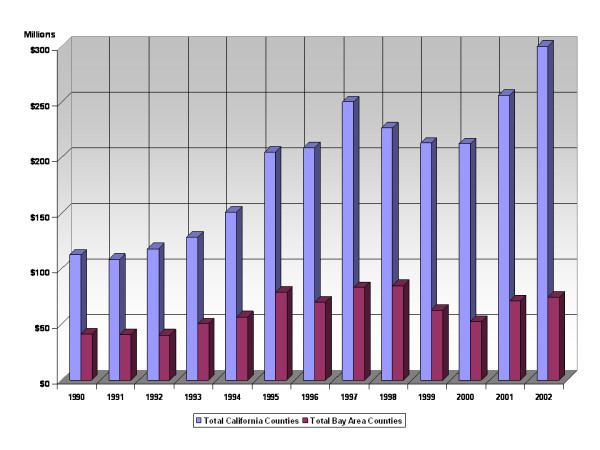
Small Business Innovation Research and Technology Awards to Silicon Valley.

During the decade of the 1990s through the early 2000s California ranked number one among states receiving federal funding for research. During this period the average annual federal obligation to California R&D in industry was approximately $6.96 billion, while support of university-based R&D averaged approximately $3.3 billion. Although the trend line points to a decreasing amount of federal spending for industry R&D in the later years of the period with an encouraging increase to universities, the fact is that federal support of California R&D nearly tripled support for university-based research. Of course, a sizeable portion, typically exceeding 50% of the total Federal R&D in California is directed toward the defense industries. But even allowing for defense spending and not taking into consideration that some biotech research is funded by the DOD, the amount of non-defense related federal funding to industry in California exceeds federal support for academic research by a considerable margin – typically by a factor of two Particularly important for the companies like Affymetrix, Symyx and other startups in the microarray field we will discuss below is the number of Small Business Innovation Research Program (SBIR) awards and Small Business Technology Transfer Program (STTR) awards going to California [Note I]. Throughout this period California has averaged around 1,000 SBIR and STTR awards, ranking first in both categories of awards, with Massachusetts typically ranking second. Moreover, during this period California has received 184 awards from the Advanced Technology Program, a program that sponsors startup companies having a university-based collaboration or academic PI. From the perspective of Silicon Valley, the 1990s were the best of times. If we compare all SBIR and STTR awards received by firms in the Bay Area zip codes that constitute Silicon Valley versus all California awards, the Bay Area has averaged 33 percent (an average of $62 million per year) of the awards with a high of 39 percent in 1993 and a low of 25 percent in 2002. Several Bay Area companies, such as Affymetrix, have been the recipients of multiple SBIR/STTR/ATP awards. Figure 3 provides an overview of SBIR and STTR awards specifically to the Bay Area.

When we consider that 58 percent of the federal funding for R&D to universities in California has gone toward funding innovation in the life sciences (see Table 2), the importance of the NIH and the Human Genome Project for the explosion of biotech firms in Silicon Valley becomes evident. Federal funding has also been significant in sustaining an entrepreneurial academic environment at Stanford and other Bay Area universities that have participated in numerous waves of technological innovation within the Silicon Valley through the students they train and the faculty engaged in research and consulting as well as in working with their university technology licensing offices to disclose, patent, and license inventions. As we have shown in another study, Stanford's openness to (in former Stanford Dean of Engineering, Jim Gibbons' phrase) "reverse engineering," the enhancement of new research directions through absorption of technological directions emerging in the Silicon Valley as key to its entrepreneurial culture, is one of the pillars of its success. Stanford receives approximately $500 million in federally funded research grants annually. Berkeley and UCSF are also in the top 20 research universities receiving federal support. As we now see, federal funding is also deeply involved in stimulating and sustaining the reverse engineering essential to this co-evolution of Bay Area research universities and the Silicon Valley.

Affymetrix was well positioned to take advantage of the flows of information from both the academic and biotech communities within Silicon Valley to acquire funding and intellectual resources necessary for assembling the pool of ideas, inventions, and know-how behind the microarray. Local university help and federal funding were essential to Affymetrix's push to begin developing the GeneChip^® ^in 1990. Encouraged by Stryer and increasingly confident about the success of the GeneChip^®^, Fodor sought to capitalize on the wave of funding for technologies associated with the NIH's goal to uncover and exploit genetic information. The Human Genome Project had been launched a few months earlier and the NIH was soliciting proposals for the development of technology in support of genomics. Ron Davis from the Stanford Biochemistry Department, together with David Botstein from Genetics, were developing technology for the human genome sequencing effort at that time. Stryer invited Davis over to Affymax to discuss the use of the gene chip technology to perform genetic sequencing by hybridization. Paul Berg, who was on the Affymax scientific board, was also interested in the technology, and he attended the meeting. Both Davis and Berg immediately saw the potential of the technology, and Davis was excited enough about what he saw to propose a collaboration with Fodor to apply for NIH funding to support the development of the gene chip. The NIH panel for sequencing technology for the Human Genome Project directed by Leroy Hood was meeting across the bay in Walnut Creek, CA in the spring of 1991, and Paul Berg arranged for Fodor to be invited to present on the peptide and DNA chip project. James Watson and a blue ribbon panel of genome scientists were in attendance, and when the meeting concluded, Fodor and Davis were encouraged to apply for funding. In September 1992 the first of several grants to Affymetrix was awarded with Stephen Fodor as PI. Co-PIs on the project were Ron Davis from Stanford and Ronald Lipschutz from Daniel H. Wagner Associates, a mathematics firm that contributed expertise on improving algorithms for sequence analysis [Note J]. The initial NIH grant, funded from 1992–95, was for $2.5 million, and together with a Phase I Small Business Innovation Research (SBIR) grant from the Department of Energy (one of several SBIR grants the company has received) for $500,000 awarded in 1992, Fodor was able to demonstrate proof of the concept of using large arrays of DNA probes in genetic analysis. A Phase II grant was awarded to assist Affymetrix in moving the technology towards commercialization. Scientists at Affymetrix also received several grants from the National Institutes of Health. For example, Fodor was principal investigator on a second round of NIH funding in 1995 for a three-year $5.5 million NIH, grant from 1995–97. One component of this grant addressed the development of chip-based sequencing, re-sequencing, sequence checking and physical, genetic, and functional mapping. A technology development component addressed the production of chips and the development of instrumentation and software specific to the chip applications.

Affymetrix's largest government award in the startup phase of the company came from the Advanced Technology Program (ATP) of the National Institute of Standards and Technology (NIST) in the Tools for DNA Diagnostics Focused Program competition in 1994. In its reports documenting the successes of its programs, the ATP lists Affymetrix as one of its banner projects [17]. The ATP program was started in 1990 to stimulate new science-based research ventures and to encourage joint ventures among universities, industry, research organizations, and consortia of companies. A consortium established by Affymetrix was awarded a $31.5 million, five-year grant in 1994 to develop miniaturized DNA diagnostic systems. Under this grant, Affymetrix directly received $21.5 million, some of which was used to fund activities at a number of collaborating institutions as subcontractors to the project. As part of this grant, Affymetrix and its partner Molecular Dynamics collaborated with researchers at the California Institute of Technology, Lawrence Livermore National Laboratory, Stanford University, the University of California at Berkeley, and the University of Washington to develop the next generation of diagnostic devices to capitalize on the advances of the Human Genome Project. After developing its core chemical synthesis technology while still funded under the ATP and SBIR grants, Affymetrix entered into agreements with OncorMed to collaborate in development of clinical validation of genetic testing services utilizing the GeneChip^® ^for analysis of genes associated with cancer; and under a separate distribution and instrumentation alliance between Affymetrix and Hewlett-Packard, Hewlett-Packard began developing and supplying a next-generation scanner to read the GeneChip^® ^in 1996. The Advanced Technology Program was particularly enthusiastic about the ways in which Affymetrix accelerated the diffusion of its technology through alliances and collaborations with the Genetics Institute, Roche Molecular Systems, Incyte Pharmaceuticals, and Glaxo Wellcome in order to continue raising capital for expanding its own internal R&D [18]. Table 3 tracks federal funding that Affymetrix received over a ten year period [Note K].

**Table 3:** Federal Funding to Affymetrix (1993–2003)

**Award Number**	**AvgAnnual (in $K)**	**Start**	**End**	**Total (in $K)**	**Government Entity**	**SBIR**	**Brief Description**
FG0392ER81275	137.5	Jul-92	May-95	550.0	Basic energy sciences (Dept. of Energy)	Y	SBIR phase I: develop spatially defined oligonucleotide arrays
R01HG000813	465.8	Sep-93	Aug-95	NA	National Human Genome Research Institute	N	The long term goals of this proposal are to construct spatially defined arrays of oligonucleotide probes and to study the feasibility of using these arrays in applications of sequencing DNA by hybridization. A multidisciplinary research program is proposed which will integrate the necessary expertise in photolithography, photochemistry, synthetic chemistry, detection technology, informatics and applications to large scale DNA sequencing.
F32HG000105	20.3	Jun-93	Aug-96	NA	National Human Genome Research Institute	N	NA
R43AI036809	37.5	Jul-94	Jan-95	75.0	National Institute of Allergy and Infectious Diseases	Y	Rapid detection of HIV drug resistance
70NANB5H1031	5,246.3	Feb-95	Jan-00	31,478	Advanced Technology Program	N	Capillary-array electrophoresis, which separates and sizes DNA fragments, for use in a compact, reusable system for use with patient blood samples in labs and hospitals. Lawrence Livermore National Laboratory, Stanford University, the University of California (Berkeley), the California Institute of Technology, and the University of Washington also will work on the project.
R43CA067604	100.0	Mar-95	Sep-95	100.0	National Cancer Institute	Y	Detection of mutations in human p53, msh2, mlh1 genes
R43DA010389	50.0	Sep-95	Mar-96	100.0	National Institute on Drug Abuse	Y	Typing human cytochrome p450 genes using DNA chips
A64472D	27.6	Jul-01	Jul-01	27.6	Human health and performance (NASA)	N	Studying the rat genome
P01HG001323	1,377.5	Sep-95	Aug-98	NA	National Human Genome Research Institute	N	Human genome sequencing and mapping with dna probe arrays
R41CA075675	49.8	Jul-97	Sep-98	99.7	National Cancer Institute	N	Genotype for radiation sensitivity in cancer patients
R43AI040400	50.0	Sep-96	Mar-97	100.0	National Institute of Allergy and Infectious Diseases	Y	Chip based genotyping of mycobacterium drug resistance
R43CA081949	133.3	Jul-99	Sep-99	133.3	National Cancer Institute	Y	Reverese engineering biological signal transduction networks
R43HD038622	50.0	Sep-00	Aug-01	100.0	National Institute of Child Health and Human Development	Y	Gene expression in endometriosis
R43HG001481	33.3	Apr-96	Oct-97	33.3	National Human Genome Research Institute	Y	Mutation screening of the human mitochondrial genome
R43NS036491	50.0	Jul-97	Jan-98	100.0	National Institute of Neurological Disorders and Stroke	Y	Cytokine message monitoring in oral tolerance
R44AI036809	250.0	Aug-95	Jul-97	372.6	National Institute of Allergy and Infectious Diseases	Y	Rapid detection of HIV 1 drug resistance
R44CA067604	244.3	Mar-96	Feb-98	359.3	National Cancer Institute	Y	Phase II of earlier project to develop a rapid and efficient method for detecting mutations on the human p53, msh2 and mlh1 genes
R44DA010389	163.9	Sep-96	Aug-98	205.5	National Institute on Drug Abuse	Y	Phase II of earlier project for typing human cytochrome p450 genes using DNA chips
R44DK053325	237.7	Jul-97	Sep-99	86.7	National Institute of Diabetes and Digestive and Kidney Diseases	Y	Genomic responses to hormone signaling
R44HG001481	249.2	Jul-97	Aug-99	368.5	National Human Genome Research Institute	Y	Continuation of mutation screening of the human mitochondrial genome project
U01HG003147	661.0	Sep-03	Jul-05	985.5*	National Human Genome Research Institute	N	Mapping sites of transcription and regulation

Two themes emerge from the way the government funded Affymetrix: the wide range of government organizations that provided the funding, and the variety of federally funded research projects at Affymetrix. The diversity of agencies that saw benefits to the GeneChip^® ^is quite apparent: the Department of Energy, NASA, and several organizations within the National Institutes of Health funded Affymetrix over the eleven-year period studied. Later affirmed by the breadth of research applications the DNA chips found, this broad set of government health organizations, such as the National Cancer Institute, the National Institute of Allergy and Infectious Diseases, and the National Institutes of Neurological Disorders and Stroke, provided early testimony to the GeneChip^®^'s widespread applicability.

These kinds of collaborative research efforts were a prerequisite to acquiring federal funding to launch the company, and they have continued ever since to be a deliberate core strategy of Affymetrix, carried over from Affymax, to maintain simultaneously within the firm an entrepreneurial as well as an academic environment. The firm's goal was to attract preeminent researchers and convince them that the company was creating cutting-edge technology. Steve Fodor was persuaded to leave his postdoctoral research position at UC Berkeley – despite his initial lack of interest in leaving academia – by the possibility of continuing to work with some the field's brightest academics as well as having in-house funding to do research. The freedom to seek outside grants to pursue research peripheral to the company's core strategies was also considered an important tool in attracting high-quality people to the project. Affymetrix has been able to attract staff who continue to keep their academic contacts through participation in grant proposals, and who have the freedom to pursue ideas to which they have dedicated their careers, while gradually migrating to a commercial environment where more tangible products can be generated. The exercise of building a consortium of other companies to work together under the ATP project, for example, fed a very collegial environment where researchers worked hard with the best people in their field around the world, pushing these technologies to a stage at which they could be commercialized successfully.

### 4. The microarray revolution: diffusion of the GeneChip^® ^and microarrays

The 1991 paper in *Science *on parallel chemical synthesis using microarrays inaugurated the field of combinatorial chemistry, and it may indeed be one of the key events in the genomics revolution. By 1999 articles in *Science *among many other scientific journals were celebrating the widespread use of microarrays and the way they had transformed genomics [19]. People who never thought they would do large-scale gene studies suddenly were eager to try their hand at monitoring thousands of genes at once. The National Institutes of Health (NIH) heavily supported this trend, funding its own microarray studies and providing grants to institutions to buy the technology. This generous support of studies using microarrays generated a flood of data that traditional journals found hard to accommodate and digital databases didn't yet know how to handle. The NIH funded workshops to spread the technology. A Cold Spring Harbor Laboratory workshop on microarrays led by Pat Brown from Stanford in 1999, for instance, was the most over subscribed laboratory course on record in the history of Cold Spring Harbor programs. The new course was not even advertised, yet eight times as many people signed up as could be accepted. Sixteen people paid $1955 each to learn how to build and use a machine for genetics research. For another $30,000, four actually took the machine home.

Research and development of microarrays was the hot new field in the 1990s. Although their approach was distinctive in focusing on *in situ *synthesis of DNA libraries on a chip, Affymetrix was not alone in the microarray field. About the same time the Affymetrix group was developing the GeneChip^®^, several academic teams were developing alternative microarray systems [Note L] [20]. Of particular importance were spotted microarrays developed at Stanford by Pat Brown, Dari Shalon, Stephen J. Smith, Mark Schena and Ron Davis. The Stanford system was a contact array that used two-color fluorescence hybridization. On the heels of this system was a non-contact array developed by Leroy Hood at Cal Tech that adapted the technology for ink-jet printers to micro spot solutions of nucleotide reagents printed on a glass substrate [21-23].

The spotted microarrays were extensions of methods that had been in use in genome analysis and molecular biology for two decades, going back to Edwin Southern's introduction of the Southern Blot [24]. Another forerunner for all the microarray work, including the work of Fodor et al., were the methods for locating the position of specific sequences in chromosomes through fluorescence in situ hybridization (FISH), which allowed cell nuclei and chromosomes to be fixed to glass microscope slides as solid support. Ron Davis had contributed to those early methods for identifying genes, and the same technique was used to fix DNA to slides as solid support for his later microarray work [25]. The technique of using ordered arrays of DNA at the core of microarray techniques also grew out of earlier work. Of special importance was the dot-blot method introduced in 1979 by Fotis Kafatos, et al., in which hybridizations were carried out in parallel and fluorescent signals representing hybridization were measured with an imaging method [26]. The procedures for constructing these arrays were manual and the spots, as in the Southern Blot method, were deposited on various types of porous filters. While effective, these early spotting methods on porous materials were not suitable for the large-scale genome analyses that took off in the 1990s: it was not possible, for instance, to reduce the size of the spots beyond certain limits, or to control their size and shape on a porous membrane. The large scale automation of these dot-blot procedures was undertaken by Hans Lehrach and his co-workers at the Berlin Max-Planck-Institute for Molecular Biology in 1994. Lehrach's group developed laboratory robotic systems for picking and spotting clones onto filters [27]. This move toward large-scale automation with robots coupled with the replacement of the porous materials used in dot-blots with impermeable supports, such as glass or silicon, were key steps in the development of the spotted microarray systems. Non-porous surfaces permitted the use of very small sample volumes and high sample concentrations of spots. Over the next few years during the early 1990s technical advances made it possible to generate arrays with very high densities of DNA spots, allowing for tens of thousands of genes to be represented in areas smaller than standard glass microscope slides. These changes to the macroscopic format of filter based arrays resulted in the miniaturized "biochip" format of the microarray that has brought about a fundamental revolution in biological analysis. By effectively making it possible to represent the entire genome of an organism on a single biochip, researchers are able to study the expression of all the genes of a particular organism at once.

The spotted microarray developed in Pat Brown's lab consisted of two principle pieces of hardware; the arrayer and scanner [28]. The arrayer was a variation of the standard "pick-and-place" XYZ-axis gantry robot common to many large university molecular biology laboratories. Glass slides coated with a poly-lysine surface were placed on a platter. The robot picks up pre-synthesized single strand or double stranded DNA samples from a 384 well microtitre plate by placing a specially designed cluster of spring-loaded printing tips into adjacent wells of the source plate, each tip filling with approximately 1 micro liter of DNA solution. The DNA samples are, in most cases, labeled by incorporating fluorescently tagged nucleotides. The cone-shaped printing tips in Brown's original system were stainless steel with manually sharpened points and a slit up the center for holding the DNA solution. They operated on the same principle as a quill pen; liquid was drawn up by capillary action and deposited when the tip made contact with the slide surface. The printing tips are tapped leaving a small (less than 0.5 nano liters) drop at identical positions on each slide. With the spacing between tips deployed in the microarrayer the entire human genome could be spotted onto a standard 1-inch by 3-inch laboratory slide.

After hybridization a fluorescent image of the array is acquired by a laser scanning confocal microscope. The scanner has a laser (or lasers) producing light at the appropriate wavelength for the excitation spectra of the two dyes (red and green) being used. The light passes through the microscope objective and illuminates a single point on the slide. The emitted light gathered by the objective is filtered to remove the excitation beam, passed through a pinhole (removing noise), and finally quantified in a photomultiplier tube. The relative amount of fluorescence is measured for each spot on the array using software Brown's team developed for segmenting the images into boxes and determining the average fluorescence for each box. The advantage of using fluorescent signals is that they do not disperse, and accordingly allow for very dense array spacing. Also a significant advantage of using two or more differently labeled probes targeted to the same spot in this system is that each can be detected separately. In this way, two-color hybridization detection allows for a direct quantitative comparison of the abundance of specific sequences between two probe mixtures that are hybridized competitively to a single array.

Brown, Shalon, and Smith [29], and Davis and Schena [30] have argued that spotted microarrays have several advantages over the in situ chips designed by Affymetrix and Edwin Southerland. As we have seen in the case of GeneChip^® ^design, in situ synthesis methods work with oligonucleotides, libraries of nucleic acid sequences of between 2–25 base pairs. On a GeneChip^® ^a given gene might be represented by 15–20 different 25-mer oligonucleotides that serve as unique sequence-specific detectors. To be effective, the Affymetrix arrays require gene sequence information for specifying the de novo synthesis of the oligomers on the array. Spotted microarrays by contrast represent genes by single DNA fragments greater than several hundred base pairs in length, and virtually any length or origin. Moreover, spotted arrays do not require prior sequence knowledge but can be produced from both known and unknown cDNA and PCR fragments. Spotted microarrays, it is argued, are more flexible and more easily adaptable to a variety of research problems in genomics. Also to the point, spotted microarrays are inexpensive by comparison to Affymetrix chips [31]. Indeed, microarrayers based on the Brown-Shalon design could basically be constructed in-house by most major university research labs at a complete cost (in 1999) of around $60,000 [32]. Brown in fact has been so committed to the low cost production of microarrayers and an open source approach as a means to expedite the production of knowledge in genomics that he posted on his Stanford website all the details of manufacture for his microarray system, including all the software updates for operation of the scanning system, details on manufacturing and servicing the printing tips, and other fine points of the system.

To study the adoption of both in situ and spotted microarray technologies, we considered the first academic articles either reporting studies based on using DNA chips or simply discussing DNA microarrays. We focused on pre-1999 studies because the DNA chip-based research began to take off in 1999. These articles broke down into four main types: results of microarray studies, overviews of how to use gene chips, technology forecasts, and descriptions of new or otherwise improved DNA chips. As an indication of what types of studies were represented in the early publications about gene microarrays, we present Table 4:

**Table 4:** Sample from Our Set of Pre-1999 DNA Chip Articles

**Title**	**Authors**	**Publication**
Towards Arabidopsis genome analysis: monitoring expression profiles of 1400 genes using cDNA microarrays	Ruan, Y; Gilmore, J; Conner, T	PLANT JOURNAL
The integration of microarray information in the drug development process	Braxton, S; Bedilion, T	CURRENT OPINION IN BIOTECHNOLOGY
Probing lymphocyte biology by genomic-scale gene expression analysis	Alizadeh, A; Eisen, M; Botstein, D; Brown, PO; Staudt, LM	JOURNAL OF CLINICAL IMMUNOLOGY
Microarrays: biotechnology's discovery platform for functional genomics	Schena, M; Heller, RA; Theriault, TP; Konrad, K; Lachenmeier, E; Davis, RW	TRENDS IN BIOTECHNOLOGY
Data management and analysis for gene expression arrays	Ermolaeva, O; Rastogi, M; Pruitt, KD; Schuler, GD; Bittner, ML; Chen, YD; Simon, R; Meltzer, P; Trent, JM; Boguski, MS	NATURE GENETICS
Analysing genetic information with DNA arrays	Case-Green, SC; Mir, KU; Pritchard, CE; Southern, EM	CURRENT OPINION IN CHEMICAL BIOLOGY
From expressed sequence tags to 'epigenomics': An understanding of disease processes	Zweiger, G; Scott, RW	CURRENT OPINION IN BIOTECHNOLOGY
Detection of heterozygous mutations in BRCA1 using high density oligonucleotide arrays and two-colour fluorescence analysis	Hacia, JG; Brody, LC; Chee, MS; Fodor, SPA; Collins, FS	NATURE GENETICS

Of the early articles we studied, by far the majority reported on the results of experiments using DNA chips. Most of these studies aimed to uncover significant genetic information in areas of existing interest, such as cancer and cardiovascular disease; to understand the role of genes already identified as being important to particular diseases; or to attempt wide-scale gene expression monitoring of organisms whose genomes were already heavily studied, such as the Arabidopsis plants and Saccharomyces yeasts. By addressing several different research communities, these initial studies served to broadcast the potential of the new microarray technology. While these studies were excellent advertisements for the technology, they also opened up promising avenues of inquiry, helping the technology establish itself in a variety of research areas.

Getting involved with the technology early on was not simply a matter of desire; generally, early authors had some affiliation with Affymetrix. In part because of their longstanding relationships and ongoing collaborations with Affymetrix and due to their internal microarray development efforts (largely arising from their collaboration on the Human Genome Project), Stanford and the NIH also possessed a great deal of in-house expertise in using microarrays, which allowed them to assist researchers from other organizations in using the technology. In fact, when we tabulated the affiliations of scientists appearing on the pre-99 microarray studies, we found that Stanford, NIH, and Affymetrix appeared most often. Table 5 lists the most frequently occurring author affiliations within the set of 130 early articles on microarrays [Note M]:

**Table 5:** First Organizations to Conduct Microarray-Based Scientific Research

**Organization**	**Authorships**
Stanford University	42
Affymetrix	41
NIH	22
Univ. Calif. Los Angeles	10
Synteni Inc.	8
Univ. Calif. Berkeley	5
Univ. Calif. San Diego	5
Roche	4
Duke University	3
Natl. Publ. Hlth. Institute, Finland	3
Tampere University, Finland	3
Univ. Calif. San Francisco	3
University of Pennsylvania	3

Perhaps unsurprisingly, the first organizations to publish studies based on research using DNA chips were often those that had the strongest links to gene chip manufacturers. In fact, the second most common organization to appear as an author affiliation among the 130 studies we surveyed published prior to 1999 was Affymetrix. The top organization was Stanford, which had collaborated extensively with Affymetrix in the development of the GeneChip^®^, was developing its on pin-based arrayers, and possessed a great deal of in-house expertise in using the gene chips. The NIH's massive network of intramural research and its strong links (including research collaborations) to Stanford and Affymetrix made it third.

It was not simply a matter of being involved with the development of various microarray systems that led to publication, later research collaborations with and between these expert organizations also coincided with earlier and more frequent publication. For example, 28% of articles with a Stanford author also had an Affymetrix scientist and researchers from Stanford spin-out Synteni (whose technology was largely based on the Dari Shalon and Pat Brown system) appeared on 20% of the Stanford publications.

**Figure 4:**
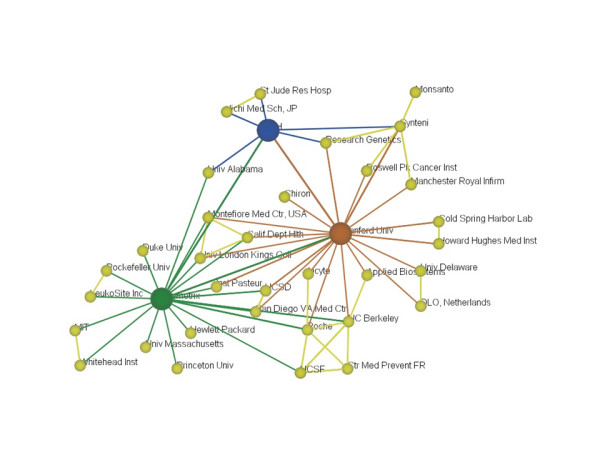
Organizational Co-Authorships from First 130 DNA Microarray Articles.

The top three organizations listed above – Stanford, Affymetrix, and the NIH – were the major "hubs" (or highly connected points) in co-authorship networks for the 130 studies we surveyed. To study the network of collaborations during this early phase of research using gene chips, we used an analysis tool that graphically places organizations according to their co-authorships with other organizations [Note N]. For example, in Figure 4, Affymetrix (large green node) co-authored with Princeton, but Princeton did not co-author with NIH (large blue node), so Princeton is near Affymetrix but distant from NIH. Furthermore, although Affymetrix and NIH co-authored papers together, they also co-authored papers with several organizations that did not co-author with both organizations, thus Affymetrix and NIH are pulled some distance apart (as opposed to NIH and Stanford, the large red node, which share more institutional co-authors) [Note O].

There were many organizations represented in the first 130 articles dealing with DNA microarrays, but Stanford, Affymetrix, and the NIH emerge as major nodes in this network. Often, other organizations would partner with one or more of these major players and then go on to collaborate with organizations previously outside the network. The heavy overlap of collaborations indicates that this was a fairly tight-knit research community. Several organizations in the center and upper right of the map collaborated with at least two of the three major players. Interestingly, many of the initial participants in microarray based research were also involved in the Human Genome Project.

Institutions that were the first to publish microarray studies and that collaborated with DNA microarray makers were also the best able to attract federal funding for microarray based research [Note P]. Organizations that appeared in Table 5 as having been the first to publish studies based on gene chip research tended to be those that received the most federal grants for DNA microarray research over the period 1993–2004 (shown in Table 6) [Note Q]. This particular phenomenon in the academic setting of being first to collaborate with Affymetrix, subsequently being first to publish DNA microarray based studies, and in turn receiving more federal funding is roughly analogous to the type of positive feedback loop that economists have used to describe how initially successful high technology firms become increasingly entrenched within their industries.

**Table 6:** Organizations Receiving the Most Federal Grants for Research Using Microarrays (1993–2004)

**Organization**	**Grants**
NIH (Intramural Grants)	108
UNIVERSITY OF CALIFORNIA	80
STANFORD UNIVERSITY	43
UNIVERSITY OF TEXAS	29
UNIVERSITY OF WASHINGTON	18
DUKE UNIVERSITY	17
UNIVERSITY OF ILLINOIS	17
UNIVERSITY OF WISCONSIN	17
EMORY UNIVERSITY	16
UNIVERSITY OF ALABAMA	16
UNIVERSITY OF MICHIGAN	16
UNIVERSITY OF PENNSYLVANIA	16
WASHINGTON UNIVERSITY	16
UNIVERSITY OF COLORADO	15
BAYLOR COLLEGE OF MEDICINE	14
JOHNS HOPKINS UNIVERSITY	14
SCRIPPS RESEARCH INSTITUTE	14
UNIVERSITY OF ARIZONA	13
UNIVERSITY OF MINNESOTA	12
YALE UNIVERSITY	12

DNA microarray makers such as Affymetrix have been hubs for an expanding network of companies and technologies across the spectrum of technologies fueling contemporary biotech, gene-based medical therapies, and areas of materials science. These companies have drawn heavily upon academic researchers as consultants and scientific advisory board members, and they have collaborated with academic researchers in sponsoring postdoctoral work and a variety of research projects funded by the NIH, NSF, DOE, and other federal agencies. The academic researchers involved have only in rare cases relinquished their university positions to move into industry. While some of these individuals, such as Schultz, Berg, Stryer, Ron Davis, Mark Davis, and others have been involved in numerous startups, they have returned to their universities (Stanford and UC Berkeley) where they have continued to develop graduate programs that incorporate these new innovations. Other Stanford faculty, such as Fabian Pease and Calvin Quate, have continued as advisors and collaborators in shaping new generations of microarray and sequencing technologies at Affymetrix. Through these technologies and the academic researchers who have participated in developing them, research programs at Stanford and other universities in a variety of different disciplines have taken new shape and direction.

In order to trace the widespread impact of microarrays on the academic research environment, Table 7 presents a chronological overview of the interest in microarrays and gene chips by several disciplines as indicated by citations to the first 130 articles published based on microarray research. (For the totals from nearly all fields citing microarray research, see Appendix A) [Note R]. The data show that interest in DNA chips and microarrays more generally was manifest in a variety of disciplines. As a new, promising, but unstable and unproven technology, microarrays were attractive as a platform that could be improved upon by many different fields. In an era when researchers were motivated to find new ways to interpret the massive amounts of data being generated by the Human Genome Initiative, researchers in just about every field of biomedicine were looking for novel high-throughput techniques to refine genetic analysis and develop tools for rapidly interpreting gene expression data. In many of the new areas, the microarray and gene chip were tools for advancing a program of "molecularizing" established disciplines. But this could not be accomplished by simply plugging in a microarray and reading off the results. New tools and even modifications of the gene chip itself had to be developed in order to assimilate the microarray to the research objectives of these several fields. Multidisciplinary teams of researchers and collaboration between academic researchers and their industry partners proved essential to advancing the technology. The demand for alternatives greatly expanded the market for these research tools and, as we show below, created opportunities for other firms to enter the market.

**Table 7:** Fields Citing Early Microarray Studies Over Time

**Field of Study**	**1991**	**1992**	**1993**	**1994**	**1995**	**1996**	**1997**	**1998**	**1999**	**2000**	**2001**	**2002**	**2003**	**2004**	**2005**	**Total**	**N**
Biochem. Research Methods	0.1%	0.3%	0.2%	0.1%	0.2%	0.9%	2.3%	4.6%	7.4%	12.1%	11.8%	16.7%	15.6%	14.0%	13.8%	100.0%	1990
Biochem. & Mol. Biology	0.0%	0.3%	0.2%	0.3%	0.3%	0.7%	2.2%	5.5%	11.8%	12.9%	14.8%	14.9%	13.6%	12.4%	10.1%	100.0%	4457
Biophysics	0.0%	0.3%	0.0%	0.3%	1.0%	0.7%	2.0%	6.0%	11.5%	11.9%	13.3%	12.4%	13.0%	12.5%	15.1%	100.0%	615
Biotech. & Appl. Microbio.	0.1%	0.2%	0.1%	0.3%	0.2%	1.0%	2.0%	6.3%	10.1%	12.7%	11.4%	14.0%	15.0%	14.5%	12.1%	100.0%	2882
Chemistry, Analytical	0.0%	0.2%	0.1%	0.1%	0.3%	1.0%	2.7%	4.6%	15.8%	14.0%	12.1%	18.0%	8.6%	12.2%	10.3%	100.0%	920
Chemistry, Physical	0.0%	0.5%	0.5%	0.5%	1.4%	1.4%	2.3%	5.6%	4.2%	11.1%	5.1%	14.4%	18.5%	19.0%	15.7%	100.0%	216
Clinical Neurology	0.0%	0.0%	0.0%	0.0%	0.0%	0.0%	1.7%	3.4%	9.3%	13.6%	14.4%	16.9%	17.8%	13.6%	9.3%	100.0%	118
Eng., Electrical & Electronic	0.0%	0.0%	0.9%	0.0%	0.0%	0.9%	0.0%	2.8%	1.9%	6.5%	0.9%	16.8%	23.4%	28.0%	17.8%	100.0%	107
Environmental Sciences	0.0%	0.0%	0.0%	0.0%	0.8%	0.0%	0.0%	3.9%	5.4%	4.7%	22.5%	19.4%	14.7%	18.6%	10.1%	100.0%	129
Genetics & Heredity	0.0%	0.2%	0.1%	0.1%	0.0%	0.9%	3.1%	5.5%	14.6%	13.7%	13.3%	13.8%	12.7%	12.3%	9.7%	100.0%	2504
Hematology	0.0%	0.0%	0.0%	0.0%	0.3%	0.0%	0.8%	1.9%	7.0%	13.6%	14.9%	18.4%	14.4%	14.6%	14.1%	100.0%	369
Immunology	0.0%	0.3%	0.0%	0.2%	0.0%	0.3%	1.5%	7.6%	4.7%	16.8%	16.0%	11.8%	14.7%	13.9%	12.3%	100.0%	619
Medical Lab Tech.	0.3%	0.3%	0.3%	0.3%	0.3%	0.6%	4.3%	8.4%	13.8%	17.9%	11.5%	13.5%	8.4%	10.7%	9.5%	100.0%	347
Medicine, General & Internal	0.0%	0.0%	1.0%	0.0%	0.0%	0.0%	4.5%	3.5%	20.3%	10.9%	13.9%	13.9%	12.9%	11.9%	7.4%	100.0%	202
Medicine, Res. & Exper.	0.0%	0.2%	0.2%	0.0%	0.2%	0.4%	5.6%	5.6%	14.7%	13.2%	18.0%	9.3%	12.2%	8.7%	11.8%	100.0%	551
Microbiology	0.0%	0.0%	0.0%	0.0%	0.0%	0.3%	1.6%	4.8%	9.4%	14.2%	17.5%	12.2%	14.3%	15.2%	10.6%	100.0%	755
Neurosciences	0.0%	0.0%	0.5%	0.0%	0.0%	0.0%	0.9%	4.9%	5.6%	12.8%	23.2%	21.1%	6.0%	16.0%	9.0%	100.0%	431
Oncology	0.0%	0.0%	0.1%	0.0%	0.0%	0.1%	1.0%	2.3%	7.1%	9.2%	14.5%	17.7%	16.7%	17.0%	14.5%	100.0%	1813
Pathology	0.0%	0.0%	0.0%	0.1%	0.0%	0.0%	0.0%	1.2%	7.3%	6.4%	16.9%	15.0%	20.2%	18.1%	14.9%	100.0%	753
Peripheral Vascular Disease	0.0%	0.0%	0.0%	0.0%	0.9%	0.0%	1.9%	3.7%	14.0%	23.4%	10.3%	18.7%	9.3%	7.5%	10.3%	100.0%	107
Pharmacology & Pharmacy	0.0%	0.0%	0.5%	0.8%	0.8%	0.8%	3.2%	4.1%	5.0%	16.8%	18.3%	13.0%	13.0%	14.5%	9.5%	100.0%	662
Physics, Applied	1.1%	1.1%	1.1%	1.1%	1.1%	2.3%	0.0%	4.5%	2.3%	3.4%	6.8%	12.5%	22.7%	20.5%	19.3%	100.0%	88
Physiology	0.0%	0.0%	0.4%	0.0%	0.0%	0.0%	0.8%	1.2%	6.1%	13.4%	18.3%	18.7%	17.5%	13.0%	10.6%	100.0%	246
Plant Sciences	0.0%	0.0%	0.0%	0.0%	0.2%	0.2%	0.2%	5.5%	7.5%	10.1%	16.4%	20.9%	14.8%	11.0%	13.3%	100.0%	602
Psychiatry	0.0%	0.0%	0.0%	0.0%	0.0%	0.0%	3.3%	20.0%	10.0%	22.2%	12.2%	4.4%	5.6%	11.1%	11.1%	100.0%	180
Public, Env. & Occup. Health	0.0%	0.0%	0.0%	0.0%	0.8%	0.8%	1.5%	2.3%	6.1%	4.6%	20.6%	22.9%	13.7%	13.7%	13.0%	100.0%	262
Statistics & Probability	0.0%	0.0%	0.0%	0.0%	0.0%	0.3%	0.0%	0.9%	1.5%	7.7%	9.9%	22.4%	21.3%	17.9%	18.1%	100.0%	648
Surgery	0.0%	0.0%	0.0%	0.0%	0.0%	0.0%	0.0%	2.4%	2.4%	4.3%	16.1%	15.2%	21.3%	21.3%	17.1%	100.0%	211
Toxicology	0.0%	0.0%	0.0%	0.0%	0.0%	0.4%	0.0%	3.1%	5.8%	19.4%	14.3%	18.2%	14.7%	11.6%	12.4%	100.0%	258
Computer Science	0.0%	0.0%	0.0%	0.3%	0.0%	0.3%	0.0%	0.9%	2.0%	7.9%	10.5%	19.2%	21.0%	19.2%	18.6%	100.0%	656
Materials Science	0.0%	0.8%	0.8%	0.0%	2.3%	1.5%	2.3%	4.6%	8.5%	6.2%	10.0%	12.3%	13.8%	15.4%	21.5%	100.0%	130
Mathematics	0.0%	0.0%	0.0%	0.0%	0.0%	0.3%	0.0%	0.8%	1.7%	7.9%	10.1%	23.6%	21.0%	16.5%	18.0%	100.0%	605
**N per year**	6	40	35	42	51	146	462	1135	2285	2973	3386	3830	3585	3447	3010		

The top three categories citing these studies (Biochemistry, Biotechnology, and Genetics) were not surprising; they represented the areas DNA microarrays were squarely targeted to address. However, the amount of interest generated around microarray research methods was quite striking. When first released there was much concern regarding the reliability of the chips, quality control issues in manufacturing them, and how to interpret results of microarray experiments. In some cases it was difficult to reproduce the results of experiments based on DNA chips. In addition, researchers discovered that each manufacturer's DNA microarray had its relative strengths and weaknesses; finding the right chip for the job was and still is of significant concern. Many studies were done both to address a particular research question and to learn something about how to better use gene chips.

The next major adopters of microarrays were those investigating cancer and cell biology. Often, these studies involved comparing the expression of thousands of genes in tumor cells to their expression in non-tumor cells. The same type of cancer (e.g. breast cancer) may involve a different (and very large) set of genes depending on the patient, so it is not enough to simply determine that gene A is related to cancer B. It is often necessary to capture the broad set of involved genes (including those regulating expression) and their interplay to begin to profile particular cancers. An understanding of the processes in cancerous cells aids in designing future drugs to disrupt the chain of events. More immediately, gene expression profiling of a particular patient's tumor through diagnostics, the genes for which are often selected by expression analysis with high density microarrays, enables prediction of the efficacy of existing treatments. Thus, microarrays enabled comparative study of gene expression in cells that led to insights about the complex processes behind cancer progression, but they also allowed for research on selecting patient-specific treatments based on gene expression profiles in tumor cells.

Some of the biological and medical fields affected by microarray research raise equally interesting issues. Microarrays enabled a broad range of researchers to better address questions such as how certain genes and their expression are related to the processes involved in particular diseases, to development and aging, and to the workings of the brain. Microarrays could also be used to address questions on evolution. In other words, microarrays not only provided a valuable tool to these researchers; in certain cases, they *made genetics more relevant *to their respective fields than it had been previously, and in particular, to their methods of inquiry.

There were also technical fields that took up research on DNA microarrays not for purposes of applying them within the field but in order to improve them and to provide better methods for interpreting gene expression data. Physicists, chemists and various kinds of engineers created custom microarrays, labeling systems for genetic material and systems for reading gene chips, or they explored new methods of manufacturing arrays. Interestingly, the sheer volume of data generated by gene expression studies forced geneticists, biologists, and others using microarrays to pull statisticians, mathematicians, and computer scientists into their research teams. Methods of reading, visualizing, and interpreting gene expression information and linking it to existing scientific knowledge became codified in a plethora of computer programs from in-house statistics and visualization tools at universities to major software suites developed by corporations that can be connected to online repositories of biological information.

In order to convey a sense of the interests in microarray technologies motivating researchers, we present in Table 8 a list of articles from many of the new fields that received a substantial number of citations. Many of these articles served as a basic bridge into a new discipline, making microarrays relevant to the science and/or vice versa. In a network view, they would represent a major forward-linking hub that collapses a question addressed within the authors' traditional field of study into a problem solvable with microarrays and motivates a flurry of subsequent research in that new domain. For example, statistical analysis of gene expression data has become a major topic of research at many universities; as the table shows, one study that used statistical methods to evaluate microarray data, despite being published in 2000, was cited over 300 times.

**Table 8:** Articles Indicating the Relevance of DNA Chips to Various Disciplines

**Field of Study**	**Authors**	**Article Title**	**Year**	**Cited**
Biochemistry & Molecular Biology	Mathews, DH; Sabina, J; Zuker, M; Turner, DH	Expanded sequence dependence of thermodynamic parameters improves prediction of RNA secondary structure	1999	1123
Biophysics	Cosnier, S	Biomolecule immobilization on electrode surfaces by entrapment or attachment to electrochemically polymerized films. A review	1999	188
Biotechnology & Applied Microbiology	Lockhart, DJ; Dong, HL; Byrne, MC; Follettie, MT; Gallo, MV; Chee, MS; Mittmann, M; Wang, CW; Kobayashi, M; Horton, H; Brown, EL	Expression monitoring by hybridization to high-density oligonucleotide arrays	1996	1556
Cardiac & Cardiovascular Systems	Stanton, LW; Garrard, LJ; Damm, D; Garrick, BL; Lam, A; Kapoun, AM; Zheng, Q; Protter, AA; Schreiner, GF; White, RT	Altered patterns of gene expression in response to myocardial infarction	2000	130
Cell Biology	Spellman, PT; Sherlock, G; Zhang, MQ; Iyer, VR; Anders, K; Eisen, MB; Brown, PO; Botstein, D; Futcher, B	Comprehensive identification of cell cycle-regulated genes of the yeast Saccharomyces cerevisiae by microarray hybridization	1998	1110
Chemistry, Medicinal	GALLOP, MA; BARRETT, RW; DOWER, WJ; FODOR, SPA; GORDON, EM	APPLICATIONS OF COMBINATORIAL TECHNOLOGIES TO DRUG DISCOVERY	1994	927
Chemistry, Physical	Collier, CP; Vossmeyer, T; Heath, JR	Nanocrystal superlattices	1998	231
Clinical Neurology	Whitney, LW; Becker, KG; Tresser, NJ; Caballero-Ramos, CI; Munson, PJ; Prabhu, VV; Trent, JM; McFarland, HF; Biddison, WE	Analysis of gene expression in multiple sclerosis lesions using cDNA microarrays	1999	122
Computer Science	Furey, TS; Cristianini, N; Duffy, N; Bednarski, DW; Schummer, M; Haussler, D	Support vector machine classification and validation of cancer tissue samples using microarray expression data	2000	237
Endocrinology & Metabolism	Kao, LC; Tulac, S; Lobo, S; Imani, B; Yang, JP; Germeyer, A; Osteen, K; Taylor, RN; Lessey, BA; Giudice, LC	Global gene profiling in human endometrium during the window of implantation	2002	106
Genetics & Heredity	Ashburner, M; Ball, CA; Blake, JA; Botstein, D; Butler, H; Cherry, JM; Davis, AP; Dolinski, K; Dwight, SS; Eppig, JT; Harris, MA; Hill, DP; Issel-Tarver, L; Kasarskis, A; Lewis, S; Matese, JC; Richardson, JE; Ringwald, M; Rubin, GM; Sherlock, G	Gene Ontology: tool for the unification of biology	2000	1273
Hematology	Melnick, A; Licht, JD	Deconstructing a disease: RAR alpha, its fusion partners, and their roles in the pathogenesis of acute promyelocytic leukemia	1999	370
Immunology	Klein, U; Tu, YH; Stolovitzky, GA; Mattioli, M; Cattoretti, G; Husson, H; Freedman, A; Inghirami, G; Cro, L; Baldini, L; Neri, AN; Califano, A; Dalla-Favera, R	Gene expression profiling of B cell chronic lymphocytic leukemia reveals a homogeneous phenotype related to memory B cells	2001	197
Infectious Diseases	Nijhuis, M; Schuurman, R; de Jong, D; Erickson, J; Gustchina, E; Albert, J; Schipper, P; Gulnik, S; Boucher, CAB	Increased fitness of drug resistant HIV-1 protease as a result of acquisition of compensatory mutations during suboptimal therapy	1999	128
Materials Science, Biomaterials	Kane, RS; Takayama, S; Ostuni, E; Ingber, DE; Whitesides, GM	Patterning proteins and cells using soft lithography	1999	262
Materials Science, Multidisciplinary	Nakagawa, M; Oh, SK; Ichimura, K	Photopatterning and visualization of adsorbed monolayers of bis(1-benzyl-4-pyridinio)ethylene moieties	2000	255
Medicine, General & Internal	Ross, R	Mechanisms of disease – Atherosclerosis – An inflammatory disease	1999	5009
Medicine, Research & Experimental	Kononen, J; Bubendorf, L; Kallioniemi, A; Barlund, M; Schraml, P; Leighton, S; Torhorst, J; Mihatsch, MJ; Sauter, G; Kallioniemi, OP	Tissue microarrays for high-throughput molecular profiling of tumor specimens	1998	916
Mycology	Goldstein, AL; McCusker, JH	Three new dominant drug resistance cassettes for gene disruption in Saccharomyces cerevisiae	1999	188
Neurosciences	Mirnics, K; Middleton, FA; Marquez, A; Lewis, DA; Levitt, P	Molecular characterization of schizophrenia viewed by microarray analysis of gene expression in prefrontal cortex	2000	243
Oncology	SolinasToldo, S; Lampel, S; Stilgenbauer, S; Nickolenko, J; Benner, A; Dohner, H; Cremer, T; Lichter, P	Matrix-based comparative genomic hybridization: Biochips to screen for genomic imbalances	1997	259
Pathology	Knuutila, S; Bjorkqvist, AM; Autio, K; Tarkkanen, M; Wolf, M; Monni, O; Szymanska, J; Larramendy, ML; Tapper, J; Pere, H; El-Rifai, W; Hemmer, S; Wasenius, VM; Vidgren, V; Zhu, Y	DNA copy number amplifications in human neoplasms – Review of comparative genomic hybridization studies	1998	339
Peripheral Vascular Disease	Hwang, DM; Dempsey, AA; Wang, RX; Rezvani, M; Barrans, JD; Dai, KS; Wang, HY; Ma, H; Cukerman, E; Liu, YQ; Gu, JR; Zhang, JH; Tsui, SKW; Waye, MMY; Fung, KP; Lee, CY; Liew, CC	A genome-based resource for molecular cardiovascular medicine – Toward a compendium of cardiovascular genes	1997	110
Pharmacology & Pharmacy	DOOLEY, CT; HOUGHTEN, RA	THE USE OF POSITIONAL SCANNING SYNTHETIC PEPTIDE COMBINATORIAL LIBRARIES FOR THE RAPID-DETERMINATION OF OPIOID RECEPTOR LIGANDS	1993	118
Plant Sciences	Reymond, P; Weber, H; Damond, M; Farmer, EE	Differential gene expression in response to mechanical wounding and insect feeding in Arabidopsis	2000	305
Spectroscopy	Yates, JR	Mass spectrometry and the age of the proteome	1998	351
Statistics & Probability	Kerr, MK; Martin, M; Churchill, GA	Analysis of variance for gene expression microarray data	2000	326
Toxicology	Waring, JF; Ciurlionis, R; Jolly, RA; Heindel, M; Ulrich, RG	Microarray analysis of hepatotoxins in vitro reveals a correlation between gene expression profiles and mechanisms of toxicity	2001	107
Virology	Martinez-Picado, J; Savara, LV; Sutton, L; D'Aquila, RT	Replicative fitness of protease inhibitor-resistant mutants of human immunodeficiency virus type 1	1999	182

While some of these articles' citations simply reflect acknowledgement of the new technology being applied in some fashion, many aim at expanding the capabilities of microarray technologies by addressing fundamental questions in an existing research domain. Nonetheless, both types of citations indicate the growing relevance of gene expression and other microarray based studies on various scientific fields.

**Figure 5:**
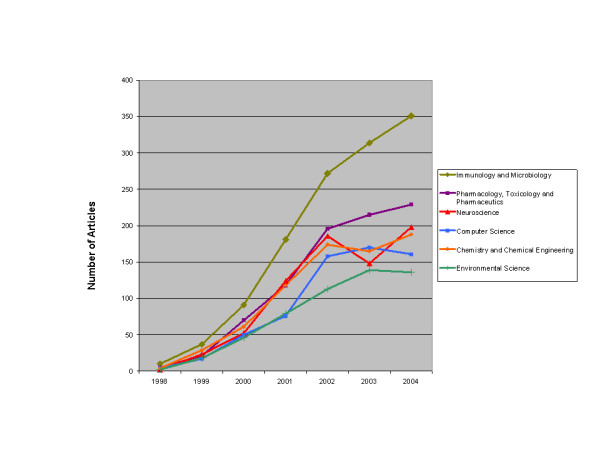
Number of Microarray Articles by Subject (1998–2004).

**Figure 6:**
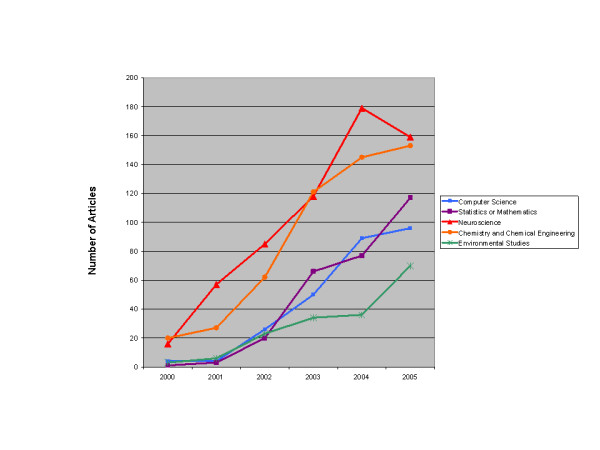
Number of Microarray Articles by Departmental Affiliation (2000–2005).

Below we chart the rising use of microarray technologies and research underpinned by microarrays through counts of microarray related studies [Note S] by subject (according to the Scopus database) and by departmental affiliation of at least one of the authors. We are particularly interested in highlighting the growth of use in particular disciplines in addition to biology, biochemistry, and genetics, and in illustrating how microarrays became relevant to a host of fields that could benefit from a better understanding of gene processes. In addition, we try to demonstrate that non-biological fields such as computer science became involved in order to enhance the microarray research itself. While neither subject classifications nor departmental affiliations provide a definitive account of the story of microarrays in these fields, we believe that the two approaches to tracking diffusion reinforce one another and at a minimum point to the growing relevance of large scale gene expression monitoring technologies in various academic disciplines. Figure 5 presents the number of microarray articles by subject over a seven year period in order to demonstrate the growing relevance of microarrays to diverse disciplines. Following, Figure 6 attempts to capture a similar picture of the spread of microarrays into different corners of academia through the departmental affiliations of authors rather than the subject classification of the article as in Figure 5.

As we have discussed above, microarrays were not a simple tool biologists and geneticists could readily apply to understanding the role of particular genes. They often had to enlist the support of colleagues in other departments to analyze, view, and interpret the data provided by DNA microarrays. In addition, research to improve numerous aspects of gene chip experiments took hold in departments outside of the biological sciences. In addition, biological fields that still had to profit significantly from the results of mapping the human genome and myriad studies on individual genes were now able to better link existing research questions to genomics questions.

### 5. Commercial interest in DNA microarray technology

Although Affymetrix has dominated the commercial market for DNA microarrays since its inception, distantly followed by Agilent, it is important to note that nearly half of scientists using microarray systems had built them locally according to plans similar to those made available by Pat Brown and his colleagues at Stanford. While these generally offered less reliability, consistency, and had fewer applications, they were far cheaper than the commercially available systems.

Alongside the research universities and other non-profit institutions that had begun in incorporating gene chips into their research programs, many companies were looking at how to enter the gene chip business and how to build complementary systems. Interestingly, many of these efforts, particularly at the smaller companies were offshoots of the university research that had begun earlier on some aspect of gene chip applications or technologies, such as bioinformatics software. Larger companies often stepped in by applying existing expertise and familiar manufacturing techniques to building their own versions of DNA microarrays.

In Table 9 we have identified organizations with a commercial interest in gene chips through their patents' backward citations to Affymetrix patents [Note U]. After finding the organizations that cited Affymetrix patents most frequently (reflecting their interest in microarray technologies), we selected those that we believed represented one of the common or important directions that microarray technologies took following the introduction of the GeneChip^® ^[Note T]. In the rightmost column we indicate whether the organization received any types of government grants for its research in this area. Twenty-five of the forty organizations listed received government grants (note the presence of universities, which depend heavily on government funding for all their research, and large companies, which rarely receive funding for this type of research). In the case of smaller, recently formed companies, 20 out of 28 received government funding. While the data is limited, it appears that new companies building technologies around microarrays were heavily supported by the federal government, helping to broaden the applications and power of the technology.

**Table 9:** Selected Organizations Frequently Citing Affymetrix Patents

**Citing Organization**	**Description of Relevant Technology**	**Grants**
3M	Photopatterned DNA chips	N
ACLARA BIOSCIENCES	Microfluidics for preprocessing of genetic material	Y
AGILENT TECHNOLOGIES	Ink-jet manufactured DNA chips	N
BIOFORCE NANOSCIENCES	Mapping nucleotide positions and molecular interactions through atomic force microscopy	Y
BIOMICRO SYSTEMS	Microfluidic systems to enhance DNA hybridization	N
CALIPER TECHNOLOGIES	Parallel microfluidic devices for preparation of genetic material	Y
CALTECH	Protein design, genetic sequence analysis and amplification, size based polynucleotide sorting	Y
CEPHEID	Rapid genetic testing for diseases and bioterrorism	Y
CLONTECH LABORATORIES	Glass, nylon, and plastic expression profiling microarrays; disease profiling arrays	Y
EASTMAN KODAK	Reagents and imaging systems for microarrays	N
FUJI PHOTO FILM	Electrochemically assembled DNA chips, optical scanners for expression arrays	N
GENE LOGIC	Analysis of Affymetrix GeneChip data	Y
GENOSPECTRA	Chronological quantitation of genetic cellular events	N
HARVARD COLLEGE	Protein arrays	Y
HYSEQ	Protein modeling and DNA chips	Y
ILLUMINA	High density optical wells and scanning systems for nucleic acid analysis	Y
INCYTE	Microarrays for gene expression studies and drug discovery	Y
KIMBERLY-CLARK WORLDWIDE	Multipurpose optical diffraction biosensors	N
LARGE SCALE PROTEOMICS	Protein identification and characterization	N
LEXICON GENETICS	Gene knockout technology to systematically discover the physiological and behavioral functions of genes	Y
MIT	Nanoparticles to create, through deposition and pattering, microelectronic devices that incorporate biological materials. Nanocrystal tagging and tracking of DNA.	Y
MAXYGEN	Protein therapy	Y
MERGEN	DNA chip maker	N
MOTOROLA	Stamped DNA chips	N
NANOGEN	Sample preparation and testing tools	Y
NANOSPHERE	Protein/Nucleic acid detection without amplification	Y
NANOSTREAM	Microfluidic systems to enhance array throughput	N
ORCHID BIOSCIENCES	Microfluidic glass chips for identity testing	Y
PERLEGEN SCIENCES	Whole genome association studies on DNA chips to better match patient genotypes with medications	Y
ROSETTA INPHARMATICS	Ink-jet microarrays and analysis software for genetic research	N
SEQUENOME	Resequencing array for genotyping	N
SOMALOGIC	Rapid protein based diagnosis of disease	Y
STANFORD UNIVERSITY	Using cell stress response to identify drug targets, nucleic acid amplification, pin based DNA chip manufacturing	Y
SYMYX TECHNOLOGIES	High throughput chemical discovery and analysis on microarrays	Y
TELECHEM INTERNATIONAL	Noncontact microspotting devices	N
THIRD WAVE TECHNOLOGIES	Molecular diagnostics and amplification	Y
UNIVERSITY OF CALIFORNIA	Flourescense tagging, expression analysis algorithms, diagnostic arrays, chemical discovery arrays	Y
UNIVERSITY OF MINNESOTA	Creating temperature gradients on DNA chips to better characterize molecular interactions	Y
VIALOGY	Data cleaning software for gene expression studies	N
ZYOMYX	Human and murine cytokine protein assays	Y

### 6. Case studies

Our case studies were chosen to explore different perspectives on the issues we have defined as salient features of the networked, symbiotic structure supporting innovation in technology regions such as the Silicon Valley; namely, the role of federal support, the ability of companies to draw upon universities to provide expertise in addressing challenging scientific questions or help them couple their existing systems to new technologies, and the ability of commercially viable technologies generated by high-tech companies to attract government funding and shape entirely new academic research directions. It might be argued that Affymetrix is a special case since, with its star-cast of consulting scientists, engineers, and successful entrepreneurs it was so remarkably positioned to take optimal advantage of the networks supporting innovation. To address such concerns we chose four case studies that represent different trajectories microarray technology could take. Affymetrix was a startup. But what about a large, well established firm with large internal resources to devote to developing its own technology for entry in the microarray market? Would it act independently of the network? Or would it draw upon the same regional networks as Affymetrix in developing its own microarray platform? What sorts of factors would motivate it to enter the market, and what sorts of resources would it draw upon? The case of Agilent, daughter firm of Silicon Valley giant Hewlett-Packard provides a striking opportunity to explore these issues.

More importantly though, these case studies will help to illustrate in detail how a viable infrastructure of scientific research and complementary technologies emerged in the case of DNA microarrays, motivating universities, industry, and government, each in different ways, to pursue competitive scientific and commercial opportunities in the emerging microarray landscape. But gene chips or DNA microarrays turn out to be only one possible application of microarrays. In the case of Symyx we explore how researchers – indeed researchers intimately connected with the original microarray project at Affymax – seized the opportunity to launch a new company that developed the basic idea of the original microarray to vigorously pursue combinatorial chemistry in the direction of non-organic materials science. Quantum Dot provides an example of a "classic" university startup coming out of an entirely different technical domain, nanocrystals, and seizing an opportunity to incorporate its technology as a component in the DNA microarray system. Our final case, Perlegen, is a spinoff of Affymetrix itself, focused on lines of research aimed at extending basic Affymetrix technology in ways directly relevant to concerns of the pharmaceutical industry. Together these case studies show that once microarrays got off the ground, players such as these made the technology an expansive and self-sustaining force.

#### 6.1 Agilent

Agilent officially entered the DNA microarray business fairly soon after Affymetrix commercially launched its GeneChip^®^s in 1998. Agilent got its start in the microarray business through a collaboration to build scanners for Affymetrix in the mid 1990s, but the company decided to compete against its business partner in 1999. Through its other bio-analysis and lab products and its connections to HP's printing and scanning business, it already housed much of the expertise necessary to create its own version of a DNA microarray and the associated hardware and software.

To get a sense of how Agilent shifted its research to address the microarray market, we searched Agilent and HP's patent portfolios for inventions pertaining to microarray systems [Note V]. After extracting the inventor names from 220 patents related to microarrays, we created patent timelines of the 140 or so inventors from these patents [Note W] to discover whether they had been at Agilent/HP or another company within the five years prior to their first microarray patent filing. Out of seventy-three inventors for whom we had the necessary data (we excluded from the analysis inventors for whom we could find no previous affiliations), sixty-one (roughly 84%) had been employees at Agilent or HP before their first patent filing on a microarray-related technology, while twelve had moved from other companies within the prior five years. Most of these employees from other companies came from biotech firms such as Applied Biosystems, Caliper, and Abaxis, with the exception of an imaging expert from Polaroid. We also uncovered two ongoing faculty consultations, one with University of Colorado's Marvin Caruthers, a well-known biochemist whose former student Douglas Dellinger had been hired at the company, and another with Karin Caldwell, a biochemical surfaces expert at the University of Utah and Uppsala University in Sweden. These hires from the biotech sector and the ongoing connections with academia may have served as means for Agilent to enhance its absorptive capacity [33], as companies such as Affymetrix have benefit significantly through collaborations with academics who help them integrate state of the art knowledge from different fields into their technology.

It also cannot be ignored that Agilent may have taken much longer in entering the business or may have never gotten started in microarrays had it not been for Affymetrix's lead. While Agilent did not receive government support for its research in this area, it did engage in many of the other formal relationships that characterize a networked innovative firm, such as partnering with Affymetrix early on for scanners and then collaborating with and investing in Rosetta Inpharmatics (founded by Stephen Friend, formerly a faculty member of Harvard Medical School; Leland Hartwell, Nobel Prize in Medicine in 2001; and Leroy Hood, then chair of the molecular biotechnology department at the University of Washington). Led by Alan Blanchard and Leroy Hood, Rosetta had devised an early ink-jet microarrayer based on Epson printers prior to its collaboration with Agilent, but partnership resulted in the use of Agilent ink-jet arrayers and Rosetta's bioinformatics software. Yet in filling the traditional role of a large company that quickly follows a startup into a new market (sometimes referred to as the "fast second"), Agilent did help to lower the costs of using microarrays and offered a new set of feature choices to consumers (such as ease of customizability) that they may not have had with a single commercial gene chip provider.

Even a company as diversified and seemingly well-positioned to entering the microarray business as Agilent still received benefits from participating in this larger network of activity surrounding high-throughput gene expression monitoring technologies. In addition to the ongoing academic consultants it retained, the company sent its researchers to numerous scientific conferences and collaborated on several papers with academics. Through early 2006, Agilent researchers had appeared as authors on over forty microarray papers, most of which were in collaboration with academic institutions such as Duke, Stanford, the University of Southern California, Michigan University, Washington University, and NC State [Note X]. Although this is common in companies with large research divisions, we believe it is an often overlooked, key source of project ideas and technical guidance. 

While Agilent hired several people to work in its newly formed microarray business, the convergence of expertise from scanners, printers, software, microfluidics, and chromatography equipment toward complete microarray systems within its own organization can be seen in the research trajectories of its scientists. Lead researchers at Agilent often acted as the central bridge gathering those with different backgrounds around microarray printers and scanning systems. These researchers themselves came from one specific field or another, such as ink-jet printing, lab instrumentation, or biochemistry, but their changing research foci can be seen in the patents they filed over years preceding Agilent's entry into the microarray business. For example, HP researcher Michael P. Caren worked in ink-jet nozzles and cartridges in the early 1990s and transitioned into creation of arrayers for genetic material by the late 90s. Many researchers exhibited a similar pattern coming from different areas of HP or outside companies and eventually coalescing around microarray technologies such as scanners, printers, and slides.

Agilent settled on a very precise ink-jet based approach to depositing strands of genetic material at specific sites. It also developed its own scanners and analysis software. Eventually, Agilent emerged as the number two microarray provider. Agilent's sixty nucleotide arrays offered very good consistency, while its microarrayers were easy to customize and provided rapid manufacture of DNA chips.

Agilent's life sciences business includes microarrays, microfluidics, gas chromatography, liquid chromatography, mass spectrometry, informatics tools, and related reagents [34]. Despite the obvious complementarities with its existing businesses and seemingly privileged entry into microarray technologies through its partnership with Affymetrix, Agilent cannot be seen as a large company forging ahead on its own. While it did have much of the expertise necessary to begin this business, Agilent still collaborated with universities and actively engaged with the scientific research community that used microarrays.

The case of Agilent unexpectedly brings forth several themes we have been exploring in the history of microarray technologies. Although the company had a great deal of the requisite expertise in-house (based on our analysis of researcher's patent histories) and was well-positioned to begin its own microarray business (particularly given its connection to Affymetrix through their scanner partnership), Agilent still found it useful to collaborate with startups, consult with universities, and engage with the larger scientific community in developing its technologies. Agilent's strong ties to the research community and the converging technical concerns of its researchers underscore the importance of multidisciplinary collaborations and a dialogue with the university-based and other scientists in developing complete systems for gene expression studies.

#### 6.2 Symyx technologies

Microarrays not only became a platform upon which various types of genomics and microbiological technologies were built; they also provided conceptual inspiration for the development of an analogous approach to non-biological materials discovery. The chemicals industry faced many of the same combinatorial chemistry challenges that plagued pharmaceuticals: a staggering number of possible molecular combinations and painstakingly slow trial and error testing processes. A method of quickly screening the candidate compounds suggested by combinatorial chemistry had been in need for years. Perhaps unsurprisingly, Peter G. Schultz, a founder of Affymax, the company that spun out Affymetrix, and at the time a professor of chemistry at Berkeley, initially conceived of the application of high-density microarray techniques to inorganic materials testing.

In the background of his first patent on a high throughput chemistry chip, Schultz and his co-inventors Xiaodong Xiang (of UC Berkeley) and Isy Goldwasser (one of his students who became President of the newly founded Symyx) explain the need for massive expansion of trial and error techniques then employed in materials discovery. Similar to the initial problem of screening drug compounds at Affymax, the inventors indicate that given the low level of scientific understanding of the properties of materials, the need for massive screening elements against combinations of other elements requires a massively parallel approach [35].

Because chemistry was insufficiently advanced to model combinations of materials and their resulting properties, it was necessary to create as many different mixtures as possible and then conduct numerous laboratory tests to determine their usefulness. The inadequate screening methods for such a large range of potential materials represented a serious bottleneck in materials discovery. The math governing how combinatorial chemistry had created a crisis of testing was clear to those in the field, as former Caltech professor Henry Weinberg, who had been hired into Symyx, and collaborator James Engstrom of Cornell write:

"Given ***n ***elements [and ***g ***elements per material], there are ***N ***= ***n!/(n-g)!g! ***possible combinations. Thus, if we restrict ourselves to 25 [ternary, ***g ***= 3] elements we need to synthesize and screen ***N ***= 25!22!3! = 2,300 ternaries. If we form these at *5% *precision [and provided ***G ***distinct compositions], the total number of compositions is approximately equal to **N **× **G **= 2,300 × 231 = 531,300 (the exact number is 399,025). One sees that even for modest precision (**Δx **= 0.05), complexity (***g ***= 3), and diversity (***n ***= 25), the numbers get very large and the experimental "boost factor" provided by high throughput combinatorial approaches is required" [36].

The number of compounds that needed to be screened against one another was simply too great using traditional methods, particularly when each process was multiplied by the need to vary temperature and pressure for each reaction [37]. Moreover, this new screening system had to have two major phases, the first in which it simply measured the reactivity of various polymers and a second, in which it another battery of tests could assess the "chemical, optical, mechanical, and electronic properties of large arrays of materials" [38].

The conceptual similarities of measuring reactions at known sites between gene chips and the new materials discovery arrays were described in a Symyx patent relating to manufacturing techniques:

The oligonucleotide probes, in turn, are available to participate in a hybridization reaction with selected nucleic acid components of the sample. Generally, this interaction of probe and sample relates to the utility of the components of the biological sample, such as the identity, concentration, purity or form of the components being sensed [39].

In comparison, in the case of Symyx arrays, an overhead infrared camera would detect how much heat had been emitted from each well, indicating to what extent the combined chemicals had reacted. However, other Symyx disclosures were quick to point out that the analogies ended at the conceptual level. Although the microarray was the essential platform that motivated the formation of Symyx, the technical challenges of implementing a materials array were quite different from those posed by a DNA chip. In the background of their early patent filings, regarding their technical approach to massive chemical screening and its dissimilarities to biological microarrays, Schultz and colleagues write:

"These solid phase synthesis techniques, which involve the sequential coupling of building blocks (e.g., amino acids) to form the compounds of interest, cannot readily be used to prepare many inorganic and organic compounds" [40].

**Figure 7:**
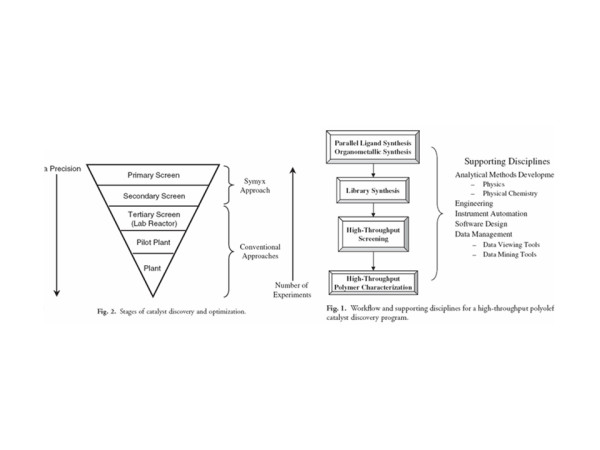
**Workflow for Chemicals Discovery and DisciplinaryUnderpinnings of Symyx High-Throughput Screening**. Figures reproduced with the kind permission of Symyx Technologies, Inc.

While the technology for building the chips was different, the overall process of screening using the microarray platform in the cases of genes and chemicals was quite similar. In their article explaining the process of materials screening employed by Symyx, Murphy et al. provide two workflows (Figure 7) outlining the underlying technologies for high-throughput screening and the ways Symyx's new on-chip techniques altered the discovery process for chemicals companies [41].

Similar to the benefits derived from using massive gene-expression monitoring technologies, the advantage to users of the technology primarily came in the form of better targets for further research (as opposed to stumbling in the dark or working with the few targets whose properties are known). In the figure on the right, Murphy et al. indicate the disciplines underpinning each part of the discovery process using Symyx arrays. This is largely analogous to the fields involved in each facet of DNA chip technology, with biology and biochemistry substituted for physics and physical chemistry. Indeed, research teams at Symyx often consist of chemists, physicists, engineers and programmers.

In addition to addressing analogous scientific and technical challenges to the GeneChip^®^, Symyx got its start in much the same way as Affymetrix. Apart from the two companies being based around a technology originally conceived of at Affymax, and sharing founders Schultz and Zaffaroni, the company maintained collaborations with academic institutions such as UC Berkeley and University Frechet and early on hired chemical engineering professor W. Henry Weinberg from Caltech to be executive vice president and chief technical officer. Kenneth J. Nussbacher, a fellow and executive vice president at Affymetrix, sits on the board of Symyx.

The company raised venture capital funding in addition to receiving a few government grants (Table 10). One government grant was aimed at discovering new materials for methanol fuel cells.

**Table 10:** Government Funding to Symyx

**Government Entity**	**Start Date**	**End Date**	**Total Funding**	**Avg FY Fund**	**Abstract**
DOD – NAVY	9/1/1998	6/1/2001	NA	1146.75	NA
DOD – NAVY	7/1/1997	1/1/1998	NA	34.5	NA
DOE – Basic Energy Sciences	8/1/1997	6/1/2000	825.382	206.346	A combinatorial approach to the synthesis and characterization of novel anode materiasantar direct methanol fuel cells

Yet the greatest source of funding for the company came from its large industrial partners with whom it has ongoing materials discovery programs. Symyx's customers, such as Celanese, Ciba, Dow, Osram OS (Siemens), and Unilever, had contributed over $85 million in near term research funding by 1999 (Table 11) [42]. Symyx has already helped to develop and a variety of materials for different purposes, including oil refining catalysts, chemicals for sensor applications, and polymers for personal care products.

**Table 11:** Licensed Symyx Discoveries

**Licensee**	**Technology**
***Commercialized Materials***	
Agfa	DirectriX needle-based detector technology for
Dow	Catalysts to produce VERSIFY™ Elastomers and Plastomers
DX-S	Computed Radiography
JSR	Polymers for Electronic Material
***Technologies in Development***	
Canon	Lab instruments
Celanese	Catalyst for Commodity Chemicals
Dow	Various Polyolefin Catalysts
Exxon Mobil	Catalyst for Commodity Chemicals
Exxon Mobil	Refining Catalyst
Hella	Automotive Oil Sensor
INEOS	Commodity Chemical Catalyst
Unilever	Personal Care Polymer
Univation	Polyethylene Monitoring Sensor
Unspecified	Catalyst for Electronic Applications
Unspecified	Sensor for Oil and Gas Exploration
Unspecified	Oral Care Polymer

Thus, Symyx's approach, which it claims is 100 times faster and 100 times cheaper than traditional research methods [43], allowed it to take the microarray platform into a variety of new sectors. In this case, it was research and licensing partnerships with established firms who had a need for materials with particular properties. Unlike Affymetrix or Agilent, which sell chips and the associated systems to customers, Symyx can be seen as more of a service provider to large companies, with its income primarily stemming from licensing royalties, using the microarray platform and a team of experts with diverse backgrounds to discover materials based on clients' needs. Although outsourcing of gene expression studies occurs regularly, it would be interesting in future research to understand why the Symyx approach has remained in house (for example, is this due to technical and/or market considerations?).

Symyx represented an important foray into new applications for the microarray platform. Developing and commercializing the technology drew upon the set of actors we have been discussing. With the key technology being developed by academics and remaining tightly woven into cutting-edge chemistry, physics, and engineering, Symyx developed a method of high throughput screening that appealed to a broad set of large customers in different industries. Its academic background and long-term research partnerships with established firms account for a great deal of the success of Symyx's discovery tools and processes.

#### 6.3 Quantum dot corporation

We now turn to the history of a startup that provided improved components for the microarray platform rather than seeking to provide technological competition for the gene chip or to prove a new application for microarrays as the organizations in the prior case studies have. In fact, this technology did not even come out of the labs of users of gene expression microarrays who wanted to enhance their performance; instead, it came from the field of quantum nanocrystals (small groupings of electrons with properties similar to an atom) that physicists and chemists had begun exploring seriously in the 1980s. Having spent years fine-tuning these crystals, but struggling to find a compelling application for them, several research teams began pursuing their use as biological labels when they realized that they were the perfect size for attaching to organic molecules. Since the GeneChip^® ^was receiving much attention at the time and many were aware that the existing fluorescent labels made it difficult to read the chips at higher densities, applying the new labeling technology in the rapidly growing DNA microarray industry was a natural choice.

In a retrospective portion of an article, Paul Alivisatos of UC Berkeley, one of the major pioneers in using quantum dots as biological labels, explains that the technology emerged from a exciting area of research for many physicists because of the ability to control the energy properties of quantum dots by changing their size. After a period of refining techniques to maintain quantum dots in solution, researchers seized upon their potential biological applications because the entire structure was roughly the size of a protein [44].

Quantum dots, groupings of free electrons that have properties very similar to atoms, had been a subject of research inquiry for chemists and physicists beginning in the 1960s. In the late 1990s, researchers at several schools, including UC Berkeley, Indiana University, and MIT, realized that quantum dots could be used to replace fluorescent dyes used in microarray studies. These dyes often did not provide sufficient contrast, particularly when genetic sequences were placed very close to each other as in high-density chips. Quantum dots offered greater clarity and because of their consistent and highly specific wavelength response, they offered potential to tag the genetic sequence with additional information. In September of 1998, Science published back-to-back articles by the competing research groups from UC Berkeley and Indiana University on the use of quantum nanocrystals as a tracking tool for biological material. As the two foundational articles explain:

"The development of nanocrystals for biological labeling opens up new possibilities for many multicolor experiments and diagnostics. Further, it establishes a class of fluorescent probe for which no small organic molecule equivalent exists. The tunability of the optical features allows for their use as direct probes or as sensitizers for traditional probes" [45].

In comparison with organic dyes such as rhodamine, this class of luminescent labels is 20 times as bright, 100 times as stable against photobleaching, and one-third as wide in spectral linewidth. These nanometer-sized conjugates are water-soluble and biocompatible [46].

Quantum dots offered dramatically smaller, clearer, and more descriptive tags for biological molecules. Because quantum dots could be tuned to a variety of wavelengths, they could be used to represent much more information than the two dyes used in microarray experiments. In fact, this tunability and signal strength offered the potential for creating gene expression tests that did not even require probes to be placed at specific sites. As explained in the background to a patent filed by the MIT quantum dot group, a particular nanocrystal could emit a wavelength corresponding to a specific gene sequence:

The system of the present invention, in contrast to fluorescently labeled probes used in the existing methods [DNA chips], is capable of not only acting as a probe for identification of a desired sequence, but is also capable of encoding information about the sequence itself. Because the inventive identification system is capable of providing both a probe and identifier, ordered arrays are not necessary for accessing genetic information, although the inventive system can still be used in traditional arrays. Instead, a collection of beads, for example, can be assembled with the desired labeled DNA fragments, wherein said beads are also encoded with information about the particular sequence. Upon binding, the oligo that hybridizes to the sample DNA can be detected by scanning the sample to identify the quantum dot labeled probe, while at the same time the sequence information can then be decoded by analyzing the quantum dot "barcode" [47].

While the initial opportunity for becoming involved with the rapidly emerging microarray market was attractive, the superior signal strength and ability to tune a probe to represent particular information had actually opened up countless tracking applications. Paul Alivisatos and his collaborators were especially eager finally to apply and commercialize their longtime basic research in the form of a biological label. When Joel Martin, a serial entrepreneur and former chemist interested in the technology visited the Berkeley Lab in 1997, the two began working out their ideas for a business, and founded Quantum Dot Corporation in 1999 [48]. QDC retained the primary investigators (Paul Alivisatos from Berkeley, Shuming Nie from Indiana, and Moungi Bawendi from MIT) from the three competing research groups as scientific advisors while hiring their former graduate students to work in its labs [Note Y]. QDC also licensed key patents for its business from Berkeley, Indiana, and MIT [49].

It is likely the widespread importance of nanocrystal tags helped Quantum Dot Corporation attract government and venture capital funding. The company received government grants before and after securing an impressive amount of venture capital funding. In early 1999, the company raised $7.5 million and in 2000 it raised an additional $30 million from several venture capital firms [50]. However, toxicity issues in the fundamental design of the tags delayed commercialization of the technology, seriously compromising its business prospects and its advantage as the first to commercialize this technology. The slowdown in the development of this promising technology prompted the government to step in with a major grant in 2004 to overhaul QDC's nanocrystals and to lower the cost of manufacturing the multipurpose labels. Table 12 shows the government grants received by Quantum Dot, including one to redesign its technology[51].

**Table 12:** Government Grants to Quantum Dot Corporation

**Funder**	**Yearly Amt. ($K)**	**Start Date**	**End Date**	**Description**
NCI	207.9	8/1/2000	2/1/2003	Development of quantum dots as tissue probes for concurrent screening of multiple markers of breast cancer
NCI	53.7	8/1/2000	7/1/2001	Enhance the sensitivity of DNA microarrays by three orders of magnitude using quantum dots, with the goal of enabling single molecule detection
ATP	1000	12/1/2004	11/30/2007	Quantum dot adoption has been slowed because they contain cadmium, which can be toxic to humans and can act as a pollutant in the environment. Quantum Dot Corporation will create new nanocrystal tags without cadmium and at the same time implement manufacturing processes that are 1000 times faster and cut costs of production by 90%.

The Federal government through the National Cancer Institute funded the development of quantum dots as an in vivo probe for breast cancer and as a better label for microarray studies, which would greatly cut down on the amount of genetic material required to conduct a study. The government also gave its most generous grant four years later through the Advanced Technology Program to develop quantum dot probes without cadmium, a potentially harmful substance in many environments. It also sought to improve QDC's manufacturing processes in order to dramatically reduce the cost of nanocrystal probes.

Quantum Dot Corporation established a variety of ongoing collaborations with universities and other companies to create new applications for its technology and build complementary lab equipment (Table 13).

**Table 13:** QDC's Corporate and University Partnerships

**Collaborator**	**Relationship**
Panasonic	Development and manufacture of instrumentation for Qdot detection
Genentech	Drug development, including imaging blood vessel tissue containing the Her-2 cancer-related gene, which is the target of Genentech's drug Herceptin
Cornell	Highly detailed imaging of blood flow using multi-photon microscopy and nanocrystals
Carnegie Mellon	Deep tissue imaging in living organisms
Vanderbilt	Nanocrystal tracking of neurotransmitters

Apart from a more typical partnership as exemplified by its collaboration with Panasonic to build necessary lab instrumentation, QDC investigated a variety of imaging applications enabled by nanocrystal tags through its collaborations with Genentech, Cornell, Carnegie Mellon, and Vanderbilt. The latter three research partnerships demonstrated that quantum dots could serve as tracking and labeling systems in many biological settings and reinforce the notion of university research changing in response to the introduction of (what had become) an industry technology.

**Figure 8:**
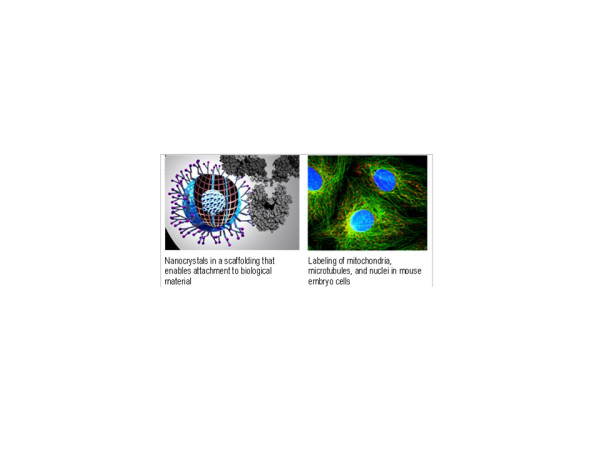
**Quantum Dots**. Images reproduced with the kind permission of Invitrogen Corporation (owner of Quantum Dot Corporation).

The story of Quantum Dot Corporation reflects that of Affymetrix in a few ways. The area of quantum dot nanotechnology was quite distant from the life sciences it eventually served before the advent of the gene chip and the enhancements to nanocrystals in the 1980s and 1990s that made them suitable as bright and reliable tags for DNA sequences and other biomolecules. While DNA microarrays provided an attractive and growing market to target, government funders, university collaborators, and industry interest prodded QDC to apply the nanocrystal tags in a variety of environments with results far exceeding those of previous labeling systems. Quantum Dot's beginnings in three universities, its ongoing ties to those institutions, and its efforts to develop its technology through collaborations with other academics demonstrate the two-way exchange we have been discussing. While the emergence of the GeneChip^® ^caused the original university physicists and chemists behind Quantum Dot to shift their research to develop particular labeling applications, the company later received input from a variety of academic experts on how to extend its basic technology into new settings. Figure 8 provides illustrations of quantum dots, their scaffoldings, and how they help to label different parts of a cell [52].

#### 6.4 Perlegen

Perlegen is our final case study in the microarray space because the company represents a significant extension of the DNA microarray into the pharmaceutical industry through the profiling of the entire genome on a set of high density chips. Perlegen has taken the GeneChip^® ^developed by Affymetrix and repurposed it specifically to address the role genetic diversity and variation can play in improved drug targeting and drug discovery.

Affymetrix spun off Perlegen to focus on whole genome scanning and providing genomics services to the pharmaceutical industry. The new company used Affymetrix's largest, highest density arrays to discover the differences in individual's DNA relevant to the treatment of a particular disease. To discover such differences, patient groups are assembled along clearly defined lines (such as the efficacy of a particular drug). Instead of using a test of a few hundred genes suspected to be involved, Perlegen's method allows for an in-depth genome-wide scan of each patient. These types of tests were the first to uncover the sets of SNPs linked to the development of particular diseases, and of more immediate importance, which genetic profiles lent themselves well to particular treatments.

Perlegen fills an important gap in the genomics space. It builds a significant bridge to the pharmaceutical industry that had previously targeted drugs at patients based on clinical diagnoses, which were often a group of symptoms or other phenotypic markers that conflated many possible genetic underpinnings of a disease. The company helps pharmaceutical firms identify sub-populations that will respond safely and more effectively to particular drugs. The ability to target drugs at specific populations based on their genetic differences allows for faster FDA approval, the resurrection of failed drugs, and the application of existing drugs to new conditions (which can be a means for filing a new patent on the compound and extending the time the drug is on patent protection). Perlegen also hopes to leverage the data it generates to conduct internal drug development.

From its beginnings, the company was closely connected to the scientific community. Affymetrix founder and CEO Steve Fodor asked David Cox, then professor of genetics and pediatrics at the Stanford School of Medicine to be chief scientific officer at the new company. Early on, Perlegen published scientific studies based on the results of its mapping of SNP markers in different populations. Cox hired his former graduate students to lead the labs and set up an environment that was characteristic of an academic research group. The company's researchers had published close to thirty scientific articles in peer-reviewed journals as of March, 2006. Within these articles, collaborations with academics from universities, other non-profit institutions, and companies were frequent (see Table 14).

**Table 14:** Perlegen Coauthorships with Selected Organizations

**Collaborator**	**Authorships**	**Articles**
UC Berkeley	4	3
Rockefeller Univ	4	3
UC San Francisco	3	2
Hyseq Pharmaceuticals	3	1
Mayo Clinic	3	1
Kyushu Univ, Japan	2	1
UC San Diego	1	1
Univ Chicago	1	1
Univ Michigan	1	1
Univ Penn	1	1
Brown Univ	1	1
University of Southern California	1	1
Pfizer	1	1
Eli Lilly & Co	1	1
Aventa Biosciences	1	1
Sugen Inc	1	1

It was important for Perlegen to attract top scientists and maintain a porous research environment in which lab members were free to collaborate with academic colleagues as needed. The multiple papers the company authored with UC Berkeley, Rockefeller, and UC San Francisco were evidence of this, and the topics ranged from cross-species genomic comparisons to a haplotype map of the human genome based on research by hundreds of International Haplotype Consortium members.

Perlegen was also similar to an academic research group and the other small companies we have been discussing in receiving a substantial amount of government funding (Table 15).

**Table 15:** Federal Funding to Perlegen

**Funder**	**Start Date**	**End Date**	**Avg FY Fund ($K)**	**Title**
National Cancer Institute	9/1/2004	8/1/2005	209.8	Comprehensive Mutational Analysis of the Cancer Genome
National Human Genome Research Institute	9/1/2004	9/1/2005	3041.8	Large-Scale Low-Cost Genotyping for the Haplotype Map
National Human Genome Research Institute	9/1/2002	6/1/2005	260.7	Evolutionary Conserved Sequences in the Human Genome
National Human Genome Research Institute	6/1/2003	3/1/2004	50.3	Comparative Microarray Sequencing of Chimpanzee genomes
National Institute of Allergy and Infectious Disease	8/1/2003	7/1/2004	250	Discovery of Salmonella signature SNPs by microarrays
National Institute of Arthritis and Musculoskeletal and Skin Diseases	9/1/2004	3/1/2005	110.9	Genetic Association of Rheumatoid Arthritis
National Institute of General Medical Sciences	5/1/1998	4/1/2003	118.6	Conserved regulatory sequences in humans and mice
National Institute of Mental Health	8/1/2004	7/1/2005	125	Genetic Association in Austism Disorder
National Institute on Aging	8/1/2004	1/1/2005	283	Genome-Wide Scan for Alzheimer's Associated Genes

The federal government also funded specific research projects at the company apart from supporting its broader goals of mapping important variations in the human genome. For example, the National Institutes of Mental Health funded a project to look at genes causing Autism, and the National Institute on Aging sponsored research to identify Alzheimer's associated genes.

In addition to the significant interest in Perlegen's technology and process by the government and academics, Perlegen's commercial potential attracted the interest of venture capital firms and pharmaceuticals seeking to establish partnerships. In fact, Perlegen raised $207 million from VC firms since its inception, an impressive amount in any sector. Pfizer also invested $50 million in the company, representing 12% ownership.

Indicating the relevance of Perlegen's approach to pharmaceutical discovery and drug targeting, its clients include Pfizer, GlaxoSmithKline, Merck, Johnson & Johnson, and AstraZeneca.

Despite its unique role in serving the pharmaceutical industry, the networked character of Perlegen, through venture capital funding, its origins in Affymetrix, the transplanting of academic researchers into its labs, and its ongoing collaborations with the scientific community demonstrates the potential for many of these different institutions to engage one another simultaneously.

## Conclusion

In this study we have explored the dynamics of innovation in a networked technology region, Silicon Valley. Our study confirms the picture put forward by a several researchers that the open character of this economy is what makes it truly innovative. In an open system innovations emerge from the network. The emergence and diffusion of microarray technologies we have traced here provides an excellent example of an open system of innovation in action. Whether they originated in a startup company environment that operated like a think-tank, such as Affymax, the research labs of a large firm, such as Agilent, or within a research university, the inventors we have followed drew heavily on knowledge resources from all parts of the network in bringing microarray platforms to light.

Another key point in our study is the role of federal funding in stimulating the innovation networks associated with the emergence and diffusion of microarray technology. Federal funding for high-tech startups and new industrial development was important at several phases in the early history of microarrays. As we have seen, federal funds were the enabling factor for several startups, and as the technology evolved, support for collaborative research projects using gene chips and microarrays was crucial to evolving the various microarray platforms and their supporting technologies. Companies developing microarray technologies such as Affymetrix and Perlegen have functioned very much like research programs at universities, and in many ways the collaborative research going on in those firms with academics is more productive and has a greater impact than research in most university settings. Federal funding of academic researchers using microarrays was fundamental to transforming the research agendas of several fields within academe. The typical story told about the role of federal funding emphasizes the spillovers *from *federally funded academic research *to *industry. Our study has shown that the knowledge spillovers worked both ways, with federal funding of non-university research providing the impetus for reshaping the research agendas of several academic fields.

In the early days of the Human Genome Project (HGP) Walter Gilbert pointed to a paradigm shift about to transform biology as a result of the efforts to sequence the genomes of all organisms and store that information in large electronic databases [53]. At the heart of this revolution were automated computer-based systems for the massive throughput of biological information and technology that would allow biologists to perform thousands of experiments in parallel. Gene sequencing technology, including PCR machines, laboratory robotic systems, such as the arrayers we have discussed, bioinformatics software such as, FASTA, BLAST, PSI-BLAST, hidden Markoff models, and other sensitive tools for imaging and interpreting sequence information: these and other tools for automated acquisition and mapping of genetic sequence information were products of the Human Genome Project, all enabling elements of the revolution in molecular biology anticipated by Gilbert. Microarrays are among the signature technologies of this ongoing Genomics Revolution.

There are important parallels between the revolution in molecular biology ushered in by the Human Genome Project and the revolution in computing and information technology presided over by the NSF and DARPA's Information Processing Technology Office in the 1960s–1970s [54,55]. Both were large scale federally funded efforts that provided support to academic research and industry startups – literally creating the field of computer science and a nascent computer industry on the one hand and the various academic, medical and commercial fields related to genomics on the other. In our view, the infrastructure of federal funding policy was crucial for enabling the Genomics Revolution. Over the span of 15 years – from 1988 through its completion in 2003 – the Human Genome Project expended more than $3.8 billion dollars in federal grants and contracts to universities, genome research centers, and industry. From the start, HGP planners anticipated and promoted private sector participation in developing and commercializing genomic resources and applications. When the HGP was initiated, vital automation tools and high-throughput sequencing technologies had to be developed or improved. The HGP leadership recognized these goals could not be achieved within the timeframe set for the Project without complementary efforts of both university and private sector researchers. The strategy succeeded beyond anyone's expectations: the cost of sequencing a single DNA base was about $10 at the outset of the HGP; by 2001, sequencing costs had fallen about 100-fold to $.10 to $.20 per base. DOE-funded enhancements to sequencing protocols, chemical reagents, and enzymes contributed substantially to increasing efficiencies and reducing costs. The commercial marketing of these technologies greatly benefited basic R&D, genome-scale sequencing, and lower-cost commercial diagnostic services. As we have shown in the case of microarrays, substantial public sector R&D investment by the DOE and the NIH launched key startup ventures such as Affymetrix, Synteni, Incyte and other firms; and as we have also shown, federal funding of research collaborations between academic and industry researchers at these companies improved the technology and reshaped the academic landscape. In addition to the HGP itself, the key policy instrument enabling these developments was the Small Business Innovation Development Act of 1982, which created the Small Business Innovation Research (SBIR) and Small Business Technology Transfer (STTR) programs [56] [Note Z]. Although not created with the Human Genome Project in mind, the Small Business Development Act and the direct federal funding of industry R&D by the NSF, NIH, and DOE provided the basis for the two-way flow of innovation between academic researchers and industry that has fueled the Genomics Revolution.

## Notes

[A] Schultz was already celebrated for his contributions to the understanding of the mechanisms of molecular recognition and catalysis in biological systems. He was working on the design of highly efficient "catalytic antibodies" able to cut, splice, and modify biological molecules at specific points. Schultz was also beginning his pioneering work on a new technique for studying proteins in which unnatural amino acids are inserted site-specifically into proteins so that their catalytic and binding properties and stability could be studied.

[B] The board included Paul Berg, Carl Djerassi, Mark Davis, Avram Goldstein and Michael Pirrung from the Stanford biochemistry, chemistry, and pharmacology departments; Murray Goodman, from biochemistry at UC San Diego; and Joshua Lederberg from Stanford and the Rockefeller University. They met weekly with Zaffaroni, Schultz, Read, and Lubert Stryer, who was the director of the board. Stryer had taken a leave of absence from Stanford to head the research team at Affymax.

[C] For an excellent discussion of Geysen's peptides on pins method and its limitations see Pirrung MC: *Molecular Diversity and Combinatorial Chemistry: Principles and Applications*. Amsterdam: Elsevier; 2004, pp. 17–20. Geysen's approach involved a strategy of iterative steps, called iterative deconvolution, in identifying the optimum set of peptide pairings that would identify a protein-binding region; the Affymax scientific board felt it involved assumptions that would allow potentially valuable drug leads to be missed.

[D] Another strategy was Richard Houghten's "tea bag" approach and the "split-and-mix" synthesis pioneered by Árpád Furka from Hungary (later at Advanced ChemTech in Louisville, KY).[1] Kit Lam from the University of Arizona (now at UC Davis) took the split-and-mix method to the next level in 1991 by growing the peptides as attachments to polystyrene beads as the supports for the synthesis. Lam's highly efficient split-and-mix synthesis method generated "one-bead one-compound" (OBOC) combinatorial peptide libraries with millions of peptides, in which each 80-μm bead displayed only one peptide entity.

[E] The ability to attach and remove molecules using light to activate or deactivate linkages at different stages of the synthesis was crucial to Pirrung and Read's ideas for in situ synthesis. The parallel synthesis in situ on the solid surface using photolithographic techniques depends on decoupling protective groups followed by coupling of oligonucleotide. In order for synthesis to progress, a protecting group on the 5'-hydroxyl terminus of the growing DNA molecule must be removed. As demonstrated by the work of Patchornik and others, this deprotection reaction is readily adapted to light control through a large class of protecting groups that are photochemically removable. With his background in photochemistry, Pirrung was deeply familiar with the research on photolabile protecting groups in mononucleotide syntheses by Patchornik and his students dating back to the early 1970s, in addition to the contributions to the field of a number of other researchers in the intervening years. For a list of more than thirty scientific publications relevant to the gene chip see Fodor SPA, Stryer L, Winkler JL, Holmes CP, Solas DW: **USPTO 5,489,678**. Photolabile nucleoside and peptide protecting groups, February 6, 1996.

[F] Pease's wife, Anna Caviani Pease was first author on the May 1994 paper in the Proceedings of the National Academy of Sciences which introduced sequencing by hybridization on the Affymetrix gene chip. Anna Pease had her Ph.D. in Chemistry from UC Berkeley in 1990 and joined Affymax shortly after. She also took a law degree from the Stanford Law School in 1995 and joined the firm of Dorsey & Whitney in the Stanford Research Park as Co-Head of the firm's Life Sciences and Healthcare, focusing on strategic aspects of patent law in the biotechnology, pharmaceutical and chemical fields. Pease continued to work at Affymetrix, where she became the chief inventor on several Affymetrix patents on DNA arrays.

[G] This includes multiple appearances by the same researcher. To identify these scientists, we extracted all of the inventors from Affymetrix patents, along with their state. We matched inventors and states to all US patents to find all their previous patents and hand classified those patents to ensure that the inventor was the same individual. While there was some overmatching and some undermatching because of common names and individuals moving across states, we believe this was a fairly complete process. We then hand-classified unique inventors based on the subject matter of the patent, which assignees they were associated with, and temporal/geographic information (e.g. you cannot file patents from two different places at the same time). In addition to identifying previous and contemporary university affiliations, we included of patents that were co-assigned to a university.

[H] Pirrung moved from Stanford to Affymetrix and then quickly moved to Duke.

By 2000, when he filed a patent with Affymetrix, Francis Collins was Director of the National Human Genome Research Initiative.

The University of California faculty who worked with Affymetrix were from the Berkeley campus.

Andrei Mirzabekov was also a professor in Russia at the Moscow Physics-Technical Institute at the time, but we chose to include his affiliation with Argonne here because that was how he became involved with Affymetrix.

The reason we say "some" of the faculty who appeared on the patents is due to our matching process. Because there were hundreds of Affymetrix inventors we decided to link them to a larger database of all patents from 1974–2003 using their name and state. We screened these resulting patents against a table of all assignees to identify potential university professors (assuming they had filed a patent at their respective institution). Following our initial matching process, we searched the web for each suspected faculty person to verify his or her affiliation. Graduate students or post docs who appeared on the patents were not included despite the fact that a few of them are now professors at other institutions.

[I] SBIR and STTR awards are for small companies with fewer than 500 employees. The PI on the award does not necessarily have to be a member of the company. For more information, see: 

[J] NHGRI Grant Number: 5R01HG000813-03

Project Title: Sequence Determination by Hybridization

Principal Investigator: Stephen A. Fodor

Abstract: The long term goals of this proposal are to construct spatially defined arrays of oligonucleotide probes and to study the feasibility of using these arrays in applications of sequencing DNA by hybridization. A multidisciplinary research program is proposed which will integrate the necessary expertise in photolithography, photochemistry, synthetic chemistry, detection technology, informatics and applications to large scale DNA sequencing. We will apply newly developed techniques in light-directed polymer synthesis to oligonucleotide chemistry, explore kinetic and solvent related parameters of target hybridization to oligonucleotide arrays, read the positions of hybridization by epifluorescence microscopy, and apply new combinatorial methods to determine sequence from the hybridization data. The method will be applied to actual sequencing applications at the yeast genome center. Successful completion of this work will lead to sequencing instrumentation that will provide order of magnitude improvements in DNA sequencing productivity and will be directly applicable to the Human Genome Project.

Institution: Affymetrix, Inc.

3380 Central Expressway

Santa Clara, Ca 95051-0704

Project Start: 25-SEP-1992

Project End: 31-OCT-1995

[K] Radius: . The asterisk* for the last item in the total column refers to the fact that Radius has not updated the data for funding received in 2004 and 2005.

[L] Edwin Southern and Uwe Maskos developed an array on impervious supports comprised of short oligonucleotides of up to 19-mer length by in situ synthesis in 1991. The method used a process of physical masking in contrast to the light directed synthetic method developed by Fodor, et al. at Affymetrix. Southern filed for a US patent on this process in 1994. See Southern EM: USPTO 5,700,637. Apparatus and method for analyzing polynucleotide sequences and method of generating oligonucleotide arrays, December 23 1997.

[M] Based on our analysis of the first 130 articles published regarding DNA chips. We used author affiliations to count institutions, thus one article could add more than one institution to these totals. The exact search query in Google Scholar (which allows for full text article searching) and Web of Science (in the TS field, with a slightly different query format): microarray OR "gene chip" OR genechip OR "DNA array" OR "oligonucleotide array" OR "DNA chip" OR "cDNA array" OR "cDNA chip" OR "oligonucleotide chip". We then found all the pre-1999 articles in Web of Science (which contains better bibliographic information for download). We were not overly concerned with finding every early microarray paper, we simply needed a sample of those papers. Moreover, it is likely that early users of microarrays were more likely to make the use of the technology a more prominent feature of the paper than later users both to highlight its novelty and to justify their approach, once the practice had become more customary. Thus, we expect that a large proportion of these early studies using microarrays explicitly referred to their methods and equipment. Approximately 60% of our search results were false positives (mostly due to "microarray") and these were eliminated from the dataset by reading the methods section of the paper (or some part of the discussion if it was a forecasting article), this left us with 130 papers. The entire search was independently checked for completeness on PubMed using the MeSH controlled terms (which added only one or two articles to our dataset) and by using Affymetrix's list of publications using DNA microarrays.

[N] We used VxInsight from Sandia Labs and its module VxOrd to do the initial placement of organizations according to their coauthorship patterns. We used the resulting coordinates in KiNG (available free from the Duke Biochemistry Department website: ) to do the final visualization.

[O] There were also several organizations that had collaborated separately but did not connect to this larger network of early research. These largely included research efforts in Korea, Taiwan, Finland, and Germany, but also included a few articles published by U.S. based authors that did not link up with the rest of the early research.

[P] Note that this distribution is heavily skewed toward 2000–2004, and continued to rise through 2004 (although we did not have complete coverage of 2004). A major uptick occurred beginning in 1999/2000, when gene chips officially hit the market. The grants in the early years were very long term grants that had microarray projects added in during later years.

[Q] Based on our searches within Radius. We believe that proposed studies making use of DNA chips have become less likely to mention the technology as it becomes more commonplace, which would mean that we capture a smaller fraction of newer studies making use of the technology as compared with older government proposals, despite the overall rising trend.

The query we used, after experimenting with the usefulness of various search terms, was: "gene chip" OR genechip OR "gene array" OR "gene microarray" OR "dna microarray" OR "dna array" OR "dna chip" OR "cdna microarray" OR "cdna array" OR "cdna chip" OR "oligonucleotide array" OR "oligonucleotide chip" OR "oligonucleotide microarray"

Washington University is based in St. Louis and is separate from the University of Washington system.

[R] Cite* (total citations from each category) reflects the number of articles in each category that cited the first 130 microarray-based studies. The articles were weighted by the number of times they cited the first 130, because we believed that this is an indication that the article is more relevant to microarrays. In addition, in most cases articles had multiple category classifications. We decided not to divide each article by the number of classifications it had because those articles with multiple classifications were more likely to be those of interest to us and, it can be argued that we should not risk downplaying the importance of an article due to the arbitrariness of a classification system. We decided to exclude categories that were not useful, such as Multidisciplinary Sciences and Multidisciplinary Chemistry.

[S] According to our keyword searches within the Scopus database. The keywords we used were similar to those above: "gene chip" OR genechip OR "gene array" OR "gene microarray" OR "dna microarray" OR "dna array" OR "dna chip" OR "cdna microarray" OR "cdna array" OR "cdna chip" OR "oligonucleotide array" OR "oligonucleotide chip" OR "oligonucleotide microarray"

[T] We considered organizations that cited Affymetrix patents fourteen or more times and used normalized assignee names to avoid undercounting because of typographical errors and acquisitions.

[U] In this case, we present the organizations that cited Affymetrix fourteen or more times, fourteen was only chosen in the interest of space. The reason we describe these organizations as "selected" is that we excluded a few organizations because the technology they developed was not very innovative or if there were already enough examples of assignees on the list that were very similar. We also excluded companies with technology that was tenuously related to DNA microarray technology. Incyte changed its name and the total number of citing patents was fifteen.

[V] We used a three step query based on keywords, classifications, and inventor names for this search. We tested this method on Affymetrix's patent and application portfolio and found 630 out of 633 of its patents. While this query method could be improved, it was not necessary for our purposes to find every single Agilent/HP patent on microarray-related systems, nor was it crucial that we exclude every single invention that was not related to microarrays.

[W] We did this by matching inventor names and state/country locations to prior patents. We made common sense assumptions such as inventors not being able to file from multiple locations on different types of technologies at the same time. We also assumed that inventors who appeared to have switched companies and then quickly moved back, or moved back and forth repeatedly, were actually different individuals (although we kept an eye out for university consultants who might have exhibited this pattern).

[X] Based on a search of the Scopus database: . The query used was (TITLE-ABS-KEY(microarray OR "gene chip" OR genechip OR "DNA array" OR "oligonucleotide array" OR "DNA chip" OR "cDNA array" OR "cDNA chip" OR "oligonucleotide chip") AND AFFIL(agilent))

[Y] Interestingly, Moungi Bawendi and Paul Alivisatos, the heads of their respective labs at MIT and Berkeley had both worked at Bell Labs in the 80s when major discoveries on the properties of quantum dots were made there.

[Z] The Small Business Innovation Development Act of 1982 legislated that 2.5 percent of the budget of any federal research program with a budget over $100 million would be devoted to assist small business concerns to obtain government contracts for their own research and development, and (in the case of the STTR Program) to assist collaboration between small business concerns and federally funded projects at universities or federally supported research centers for the purpose of transferring the technology to the commercial sector.

**Appendix A:** Total Citations to Early Microarray-Based Studies by Field

**Field of Study**	Cite*	**Field of Study**	Cite*
Biochemistry & Molecular Biology	4474	Medical Informatics	82
Biotechnology & Applied Microbiology	2889	Agronomy	71
Genetics & Heredity	2509	Ophthalmology	70
Biochemical Research Methods	1997	Dentistry, Oral Surgery & Medicine	70
Oncology	1822	Radiology, Nuclear Medicine & Medical Imaging	69
Cell Biology	1507	Computer Science, Information Systems	69
Chemistry, Analytical	920	Veterinary Sciences	69
Pathology	759	Agriculture, Dairy & Animal Science	57
Microbiology	756	Nutrition & Dietetics	51
Pharmacology & Pharmacy	664	Transplantation	48
Computer Science	663	Horticulture	48
Statistics & Probability	658	Electrochemistry	48
Immunology	621	Otorhinolaryngology	47
Biophysics	616	Evolutionary Biology	45
Mathematics	612	Parasitology	45
Plant Sciences	605	Optics	43
Medicine, Research & Experimental	553	Computer Science, Theory & Methods	42
Neurosciences	431	Geriatrics & Gerontology	39
Hematology	370	Anatomy & Morphology	38
Medical Laboratory Technology	354	Automation & Control Systems	37
Biology	301	Substance Abuse	36
Virology	278	Polymer Science	36
Public, Environmental & Occupational Health	270	Physics, Condensed Matter	36
Toxicology	259	Pediatrics	36
Endocrinology & Metabolism	254	Information Science & Library Science	34
Physiology	246	Marine & Freshwater Biology	31
Chemistry, Physical	217	Critical Care Medicine	30
Surgery	213	Zoology	30
Infectious Diseases	206	Ecology	28
Urology & Nephrology	203	Orthopedics	28
Medicine, General & Internal	202	Physics	26
Chemistry, Organic	184	Spectroscopy	25
Psychiatry	180	Allergy	22
Chemistry, Medicinal	167	Microscopy	18
Developmental Biology	165	Materials Science, Biomaterials	17
Gastroenterology & Hepatology	160	Behavioral Sciences	16
Environmental Sciences	133	Computer Science, Hardware & Architecture	16
Materials Science, Multidisciplinary	130	Medicine, Legal	16
Engineering, Biomedical	127	Mechanics	16
Obstetrics & Gynecology	123	Agriculture, Multidisciplinary	14
Food Science & Technology	120	Engineering, Environmental	14
Clinical Neurology	118	Health Care Sciences & Services	14
Reproductive Biology	117	Integrative & Complementary Medicine	14
Respiratory System	116	Computer Science, Cybernetics	13
Computer Science, Artificial Intelligence	115	Materials Science, Coatings & Films	13
Engineering, Electrical & Electronic	113	Sport Sciences	13
Instruments & Instrumentation	108	Physics, Mathematical	13
Peripheral Vascular Disease	107	Physics, Fluids & Plasmas	13
Mycology	89	Rehabilitation	12
Cardiac & Cardiovascular Systems	89	Crystallography	12
Physics, Applied	88	Nuclear Science & Technology	12
Rheumatology	88	Mathematics, Applied	11
Chemistry, Applied	87	Agriculture, Soil Science	10
Dermatology	86	Gerontology	10

**Appendix B:** Organizations Citing Affymetrix Patents

**Assignee**	**Cite**	**Assignee**	**Cite**
AFFYMETRIX, INC.	3248	SOMALOGIC, INC.	20
AGILENT TECHNOLOGIES, INC.	402	BIOFORCE NANOSCIENCES, INC.	20
LEXICON GENETICS INCORPORATED	292	MASSACHUSETTS INSTITUTE OF TECHNOLOGY	20
~Individually Owned Patent	133	SCYNEXIS CHEMISTRY & AUTOMATION, INC.	20
ZYOMYX, INCORPORATED	126	ISIS PHARMACEUTICALS, INC.	19
LARGE SCALE PROTEOMICS CORP.	123	YALE UNIVERSITY	19
SYMYX TECHNOLOGIES, INC.	106	PACKARD BIOSCIENCE CORPORATION	19
ROSETTA INPHARMATICS, INC.	91	HYSEQ, INC.	18
IRORI	90	LJL BIOSYSTEMS INC.	17
UNIVERSITY OF MINNESOTA, THE REGENTS OF	89	GENOSPECTRA, INC.	17
MOTOROLA, INC.	87	APPLERA CORPORATION	17
CALIFORNIA INSTITUTE OF TECHNOLOGY	77	3 M INNOVATIVE PROPERTIES COMPANY	16
VIALOGY CORPORATION	77	GENE LOGIC, INC.	16
UNIVERSITY OF CALIFORNIA, THE REGENTS OF	73	LINDEN TECHNOLOGIES, INC.	16
SEQUENOM, INC.	72	ARCTURUS ENGINEERING, INC.	16
MAXYGEN, INC.	67	THIRD WAVE TECHNOLOGIES, INC.	16
AFFYMAX TECHNOLOGIES N.V.	60	CORNING INCORPORATED	15
CLONTECH LABORATORIES, INC.	47	DUKE UNIVERSITY INC.	14
DISCOVERY PARTNERS INTERNATIONAL, INC.	46	SIGNATURE BIOSCIENCE, INC.	14
UNIVERSITY OF HOUSTON	45	CANON KABUSHIKI KAISHA	14
CEPHEID	44	PROTOGENE LABORATORIES, INC.	14
AMERSHAM BIOSCIENCES AB	43	CHRYSALIS TECHNOLOGIES, INCORPORATED	14
METRIGEN, INC.	39	PERLEGEN SCIENCES, INC.	14
HARVARD COLLEGE, PRESIDENT AND FELLOWS	39	UNITED STATES OF AMERICA, NAVY	14
ILLUMINA, INC.	38	LARGE SCALE BIOLOGY CORPORATION	13
NANOGEN, INC.	38	EOS BIOTECHNOLOGY, INC.	13
NOVARTIS AG (FORMERLY SANDOZ LTD.)	37	UNIVERSITY OF NORTH CAROLINA	13
CALIPER TECHNOLOGIES CORP.	37	WHITEHEAD INSTITUTE FOR BIOMEDICAL RESEARCH	13
ORCHID BIOSCIENCES, INC.	36	CELLOMICS, INC.	13
OLIGOS ETC. INC.	36	PROLUME, LTD.	13
BURSTEIN TECHNOLOGIES, INC.	34	CURAGEN CORPORATION	12
BIOMICRO SYSTEMS, INC.	34	HIGH THROUGHPUT GENOMICS, INC.	12
DAVID SARNOFF RESEARCH CENTER, INC.	30	NUGEN TECHNOLOGIES, INC.	12
BATTELLE MEMORIAL INSTITUTE	30	ABLE SIGNAL COMPANY LLC	12
FUJI PHOTO FILM CO., LTD	29	BECKMAN COULTER, INC.	12
EASTMAN KODAK COMPANY	28	GLAXO WELLCOME INC.	12
KIMBERLY-CLARK WORLDWIDE, INC.	26	HANDYLAB, INC.	11
AVIVA BIOSCIENCES CORPORATION	26	ACLARA BIOSCIENCES, INC.	11
UNIVERSITY OF TEXAS	26	BOSTON UNIVERSITY	11
PICOLITER INC.	26	HEWLETT-PACKARD COMPANY	11
APPLIED GENE TECHNOLOGIES, INC.	24	IBM CORPORATION	11
WISCONSIN ALUMNI RESEARCH FOUNDATION	24	HOWARD HUGHES MEDICAL INSTITUTE	11
SARNOFF CORPORATION	23	PHYLOS, INC.	11
STANFORD UNIVERSITY	23	INGENEUS CORPORATION	10
NANOSPHERE, INC.	23	PRINCETON UNIVERSITY	10
NANOSTREAM, INC.	23	QIAGEN GENOMICS, INC.	10
NYXIS NEURO THERAPIES, INC.	22	PROMEGA CORPORATION	10
VERIFICATION TECHNOLOGIES, INC.	21	UNIVERSITY OF MICHIGAN	10
THOMAS JEFFERSON UNIVERSITY	20	STMICROELECTRONICS S.R.L.	10
CORNELL RESEARCH FOUNDATION INC.	20	TELECHEM INTERNATIONAL INC.	10
MERGEN, LTD.	20	EPOCH BIOSCIENCES, INC.	10

Cite reflects the number of times all of the organizations patents cited Affymetrix patents.
